# ATM Promotes RAD51-Mediated Meiotic DSB Repair by Inter-Sister-Chromatid Recombination in *Arabidopsis*

**DOI:** 10.3389/fpls.2020.00839

**Published:** 2020-06-25

**Authors:** Yuan Yao, Xiaojing Li, Wanli Chen, Hui Liu, Limin Mi, Ding Ren, Aowei Mo, Pingli Lu

**Affiliations:** ^1^School of Life Sciences, Fudan University, Shanghai, China; ^2^State Key Laboratory of Crop Stress Adaptation and Improvement, Key Laboratory of Plant Stress Biology, School of Life Sciences, Henan University, Kaifeng, China

**Keywords:** meiosis, recombination, DSBs, inter-sister chromatids, ATM, RAD51

## Abstract

Meiotic recombination ensures accurate homologous chromosome segregation during meiosis and generates novel allelic combinations among gametes. During meiosis, DNA double strand breaks (DSBs) are generated to facilitate recombination. To maintain genome integrity, meiotic DSBs must be repaired using appropriate DNA templates. Although the DNA damage response protein kinase Ataxia-telangiectasia mutated (ATM) has been shown to be involved in meiotic recombination in *Arabidopsis*, its mechanistic role is still unclear. In this study, we performed cytological analysis in *Arabidopsis atm* mutant, we show that there are fewer γH2AX foci, but more RAD51 and DMC1 foci on *atm* meiotic chromosomes. Furthermore, we observed an increase in meiotic Type I crossovers (COs) in *atm.* Our genetic analysis shows that the meiotic phenotype of *atm rad51* double mutants is similar to the *rad51* single mutant. Whereas, the *atm dmc1* double mutant has a more severe chromosome fragmentation phenotype compared to both single mutants, suggesting that ATM functions in concert with RAD51, but in parallel to DMC1. Lastly, we show that *atm asy1* double mutants also have more severe meiotic recombination defects. These data lead us to propose a model wherein ATM promotes RAD51-mediated meiotic DSB repair by inter-sister-chromatid (IS) recombination in *Arabidopsis*.

## Introduction

Meiosis is a fundamental biological process during sexual reproduction in eukaryotes that generates haploid gametes (sperms and eggs) in preparation for fertilization. To successfully complete meiosis, a series of homologous chromosome interactions are required, including paring, synapsis and meiotic recombination (Page and Hawley, 2003). Meiotic recombination, specifically crossovers (COs), ensures accurate homologous chromosome segregation during the first meiotic division. Meiotic recombination is initiated by the formation of DNA double strand breaks (DSBs) which are catalyzed by the conserved topoisomerase-like protein SPO11 (Grelon et al., 2001; Stacey et al., 2006). These DSBs are processed by the MRN (MRE11-RAD50-NBS1) complex to create 3′single-stranded DNA (ssDNA) overhangs (Bleuyard et al., 2004; Waterworth et al., 2007; Samanic et al., 2013). The ssDNA is bound by two homologs of bacterial RecA protein, RAD51 and DMC1, forming nucleoprotein filaments that facilitate searching for homologous DNA templates for repair (Vignard et al., 2007; Goldfarb and Lichten, 2010). During meiosis, DSB repair preferentially utilizes homologous chromosomes as repair templates to produce COs or non-crossovers (NCOs) (Hunter and Kleckner, 2001; Shrivastav et al., 2008). The number and distribution of meiotic COs per meiocyte is regulated to ensure accurate homologous chromosome segregation (Jones and Franklin, 2006). However, the number of DSBs usually substantially exceeds the number of COs in each meiosis (Pan et al., 2011; Serrentino and Borde, 2012). There are ∼150–250 DSBs in each *Arabidopsis* meiosis, but only ∼9–10 COs (Copenhaver et al., 1998; Serrentino and Borde, 2012; Choi et al., 2018). *Arabidopsis* has at least two types of COs: Type I and Type II (Copenhaver et al., 2002). Type I COs, which comprise ∼85% of total COs, are interference sensitive and depend on ZMM proteins (MLH1, HEI10, et al.); whereas Type II COs (∼15%) are interference insensitive and rely on MUS81 (Copenhaver et al., 2002; Mercier et al., 2015). CO interference is a poorly understood phenomenon that prevents closely spaced double COs (Berchowitz and Copenhaver, 2010). DSBs that are not repaired as COs are repaired by inter-homolog recombination as NCOs or inter-sister-chromatid recombination (Lu et al., 2012; Wijnker et al., 2013).

The DNA damage response kinase, Ataxia-telangiectasia mutated (ATM) is a central regulator of DNA damage response pathways. DSBs signals activate ATM which in turn phosphorylates numerous effector proteins for DNA damage responses in somatic cells (Marechal and Zou, 2013). ATM also has multiple roles in meiosis (Shiloh and Ziv, 2013; Udayakumar et al., 2015). In yeast and mouse, ATM negatively regulates meiotic DSB formation and distribution (Lange et al., 2011; Mohibullah and Keeney, 2017). ATM can also suppress the formation of extra meiotic COs (Barchi et al., 2008; Anderson et al., 2015). Interestingly, in mouse, ATM deficiency results in an increase of Type I COs (Barchi et al., 2008). In contrast, the deletion of the yeast ATM homolog Tel1 results in an increase of Type II COs (Anderson et al., 2015). ATM is also required to implement the meiotic checkpoint response in yeast (Penedos et al., 2015) and for phosphorylation of the DNA damage related histone H2AX during meiosis in mouse and yeast (Stucki and Jackson, 2006; Widger et al., 2018). However, whether or not *Arabidopsis* ATM also has similar meiotic functions is unclear.

Studies in yeast revealed that ATM (Tel1) promotes inter-homolog (IH) recombination and suppresses RAD51-mediated inter-sister (IS) recombination by phosphorylating Hop1 with the assistance of Mek1 and other proteins during meiosis (Wan et al., 2004; Niu et al., 2007; Carballo et al., 2008; Chuang et al., 2012; Humphryes and Hochwagen, 2014). Consistent with these observations, the *Arabidopsis* Hop1 homolog, ASY1, promotes DMC1-mediated IH repair rather than RAD51-mediated IS repair (Sanchez-Moran et al., 2007; Kurzbauer et al., 2012; Da Ines et al., 2013). In yeast, the *atm* (*tel1*) mutant does not exhibit meiotic recombination defects (Cartagena-Lirola et al., 2006). However, in multicellular organisms, such as mouse, fly and *Arabidopsis, atm* mutants show DSB repair defects (Xu et al., 1996; Garcia et al., 2003; Jones et al., 2012). However, how ATM regulates the balance between IH/IS repair in meiosis is still not clear. Here, in *Arabidopsis*, we combine genetic and cytological analyses to investigate how ATM regulates meiotic recombination, demonstrating that ATM promotes the RAD51-mediated meiotic DSB repair by inter-sister-chromatid recombination.

## Results

### Isolation and Identification of a Novel *atm* Mutant

**FIGURE 1:**
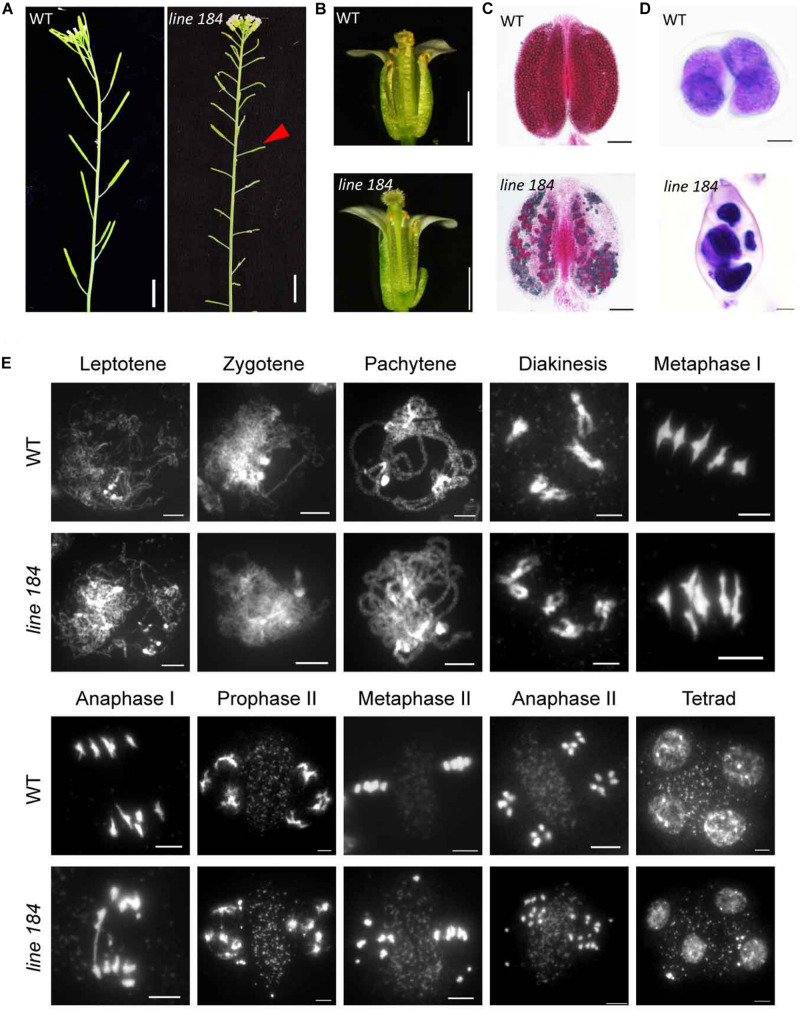
Phenotypic analysis of a mutant allele with fertility defects. **(A)** Plant fertility phenotype of wild type (L*er*) and *line 184* mutant. Red arrow indicates short silique of the mutant. Bar = 1 cm. **(B)** Morphology of open flower of wild type (L*er*) and *line 184* mutant. The mutant has shorter filaments and nearly no pollen grains could be observed on the stigma. **(C)** Pollen viability (Alexander staining) in wild type and *line 184* mutant anthers. Bar = 100 μm. **(D)** Morphology of microspore tetrad (stained with toluidine blue). Normal meiosis produce tetrad contains four microspores whereas the mutant produce polyads contain more than four microspores. Bar = 10 μm. **(E)** Chromosome morphology of male meiosis in wild type and *line 184*. Bar = 5 μm.

To identify genes that regulate meiotic recombination, we screened a Ds insertion library generated in Landsberg *erecta* (L*er*) *Arabidopsis* (Sundaresan et al., 1995), seeking sterile plants with meiotic defects. We found a sterile line, designated 184. The mutant has normal vegetative growth, but shorter siliques compared with that of wild type (Figure 1A). In contrast to wild type flowers, the mutant does not shed pollen grains onto its stigma (Figure 1B). Alexander staining revealed that about 90% pollen (viable/total: 41/445, *n* = 34) is non-viable in the mutant (Figure 1C). Light microscope imaging of Toluidine Blue-stained tetrad-stage meiocytes showed that 82% of meiocytes in the mutant produce polyads after meiosis, compared to the four microspores observed in wild type (Figure 1D), indicating a defect in male meiosis. We crossed heterozygous line 184 (female) with wild type to produce an F1 heterozygote which we then allowed to set self-fertilized F2 seed. The F2 progeny plants had a ∼3:1 segregation ratio for fertile (104) and sterile (36) individuals (*P* = 0.89, chi-square test), indicating that the mutation is a recessive allele.

To determine whether the male meiotic defects in the mutant are associated with aberrant chromosome morphologies, we stained chromosome spreads with 4′, 6-diamidino-2-phenylindole (DAPI). Chromosomes from wild type and mutant meiocytes exhibit no obvious morphological differences up to diakinesis (Figure 1E). Similar to former reported *atm-1* mutant, we did not observe any univalents in diakinesis cells (*n* = 51) of *atm-5* (Garcia et al., 2003). At metaphase I, 29% of mutant meiocytes (*n* = 38) have two early separated homologous chromosomes, whereas wild type metaphase I meiocytes consistently have five bivalents (Figure 1E). In wild type anaphase I, bivalents separate and 5 pairs of intact univalents segregate to opposite poles, and in anaphase II sister chromatids segregate to form four groups of 5 chromosomes. Mutant meiocytes, by comparison, have fragmented chromosomes in 94% of anaphase I meiocytes, and 93% of anaphase II meiocytes yield abnormal polyads with unequal amounts of DNA (Figure 1E). These results suggest that the mutant has meiotic DSB repair defects.

**FIGURE 2:**
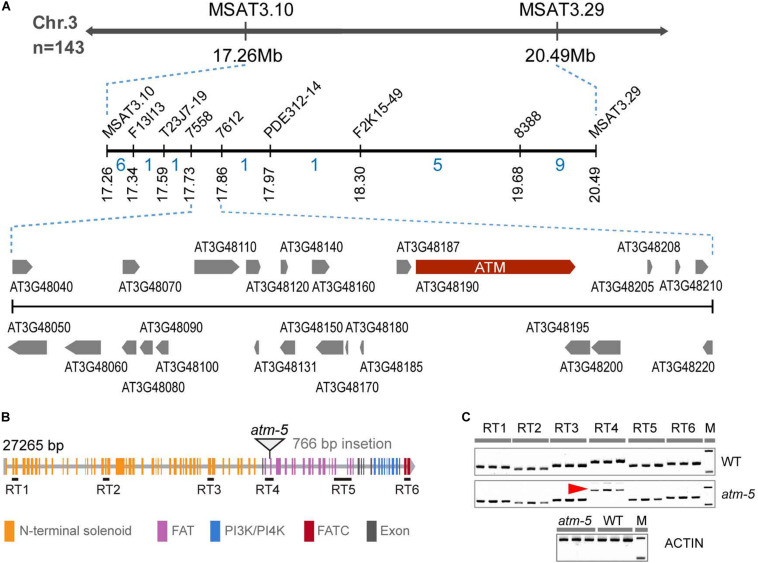
Identification of the mutated gene in the mutant. **(A)** The fine-linkage map generated by analyzing 143 *line 184* × Col-0 F2 segregating plants. The recombinant numbers are given between markers. **(B)** Diagram of *ATM* gene, the triangle showing the position of 766 bp insertion. All the colored rectangles represent an exon and each color indicates gene coding regions of corresponding protein domains. **(C)** RT-PCR detection of *ATM* transcripts. Six pairs of primers (30 cycles of PCR) were used to analyze the transcript and the location of these primer pairs was shown in **(B)**. The red arrow indicated the abnormal transcription products of *ATM* detected in *atm-5*. Amplification of the *ACTIN* cDNA has been used as a control (25 cycles of PCR).

To identify the mutated gene, we constructed an F2 mapping population for map-based cloning. We mapped the mutation to a 370 kb interval on chromosome 3 which includes the *ATM* locus (Figure 2A). The fertility and chromosome fragmentation phenotypes of our mutant are consistent with the known meiotic phenotypes previously described for *atm* (Garcia et al., 2003). After cloning and sequencing the entire *ATM* genomic region in our mutant, we found a 766 bp insertion in the intron-exon junction of the 49th exon (Figure 2B and Supplementary Figure S1A). We used a complementation test to validate the identity of our mutant as *ATM*. We crossed a heterozygous *line 184* plant with the previously characterized *atm-2* T-DNA allele (Garcia et al., 2003) to generate F1 progeny. In the F1 population, we observed a phenotypic segregation of ten fertile and eight sterile plants. The ATM genotypes correlated with their phenotypes. Cytological analysis further revealed that *line 184*/*atm-2* plants also exhibit chromosome fragmentation phenotypes during meiosis (Supplementary Figure S1C). Taken together, we conclude that the mutated gene in the *line 184* is *ATM*. Following the naming convention of the prior alleles (Garcia et al., 2003; Waterworth et al., 2007; Inagaki et al., 2009), we call our allele *atm-5*.

To investigate how the insertion affects *ATM* expression, we designed six pairs of primers corresponding to distinct regions of the coding sequence to detect transcripts by RT-RCR in both wild type and *atm-5* (Figure 2B and Supplementary Table S1). All regions had detectable transcripts in mutant and wild type plants, suggesting the *ATM* mRNA is not truncated in *atm-5.* However, the band corresponding to RT4, which includes the insertion site was larger in *atm-5* than in wild type (Figure 2C). Cloning and sequencing *ATM* cDNA from *atm-5* and wild type revealed that the insertion was integrated into the *ATM* mRNA in *atm-5* (Supplementary Figure S2A), potentially due to abnormal splicing. The integrated sequence generates a premature stop codon in the *atm-5* mRNA, leading to a truncated protein (Supplementary Figure S2B) lacking its kinase domain (FAT, PI3/4K, and FATC).

### There Are Fewer γH2AX Foci in *atm* Meiocytes

**FIGURE 3:**
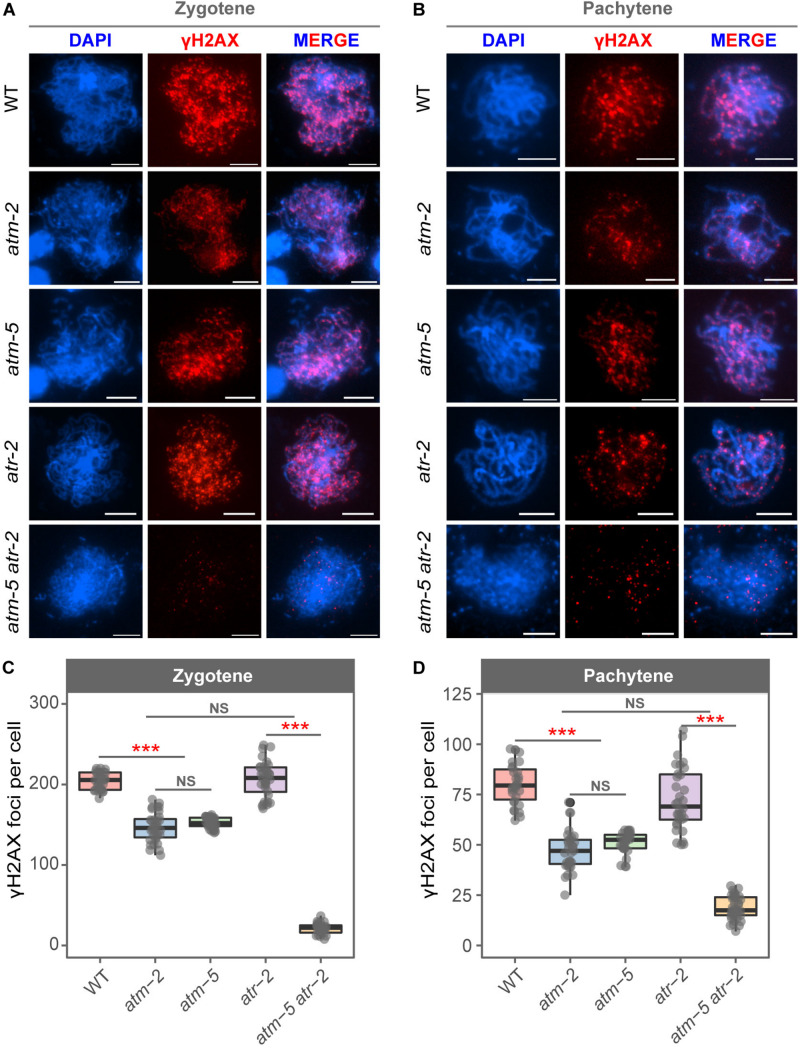
Immunolocalization of γH2AX in male meiocytes of different alleles. **(A)** γH2AX localization on zygotene stage chromosomes of wild type, *atm-2*, *atm-5*, *atr-2*, and *atm-5 atr-2* respectively. **(B)** γH2AX localization on pachytene stage chromosomes of wild type, *atm-2*, *atm-5*, *atr-2*, and *atm-5 atr-2* respectively. **(C)** Number of γH2AX foci per cell in zygotene stage in different alleles: wild type (204.07 ± 10.64, *n* = 30), *atm-2* (146.84 ± 18.55, *n* = 32, *P* = 1.40E-11), *atm-5* (152.3 ± 6.59, *n* = 30, *P* = 2.91E-11), *atr-2* (208.48 ± 24.83, *n* = 33, *P* = 4.36E-1) and *atm-5 atr-2* (21.1 ± 6.58, *n* = 29, *P* = 4.31E-11). **(D)** Number of γH2AX foci per cell in pachytene stage in different alleles: wild type (80.2 ± 10.31, n = 30), *atm-2* (47.31 ± 10.02, *n* = 31, *P* = 6.27E-11), *atm-5* (51.07 ± 5.33, *n* = 30, *P* = 2.93E-11), *atr-2* (72.44 ± 15.66, *n* = 33, *P* = 1.86E-2) and *atm-5 atr-2* (18.64 ± 5.86, *n* = 36, *P* = 3.64E-12). Bar = 5 μm. NS: *P* > 0.05, ***P < 0.001 (Wilcoxon Rank Sum test). Cells counted in each allele are from at least 30 plants. The *atm-2* and *atm-5* are two independent *atm* mutant alleles.

The phosphorylated histone H2AX (γH2AX) is deposited at DNA damage sites, and is commonly used as a molecular marker of DSBs (Lowndes and Toh, 2005). Mouse ATM is required for the formation of γH2AX during meiosis (Bellani et al., 2005; Widger et al., 2018). However, whether *Arabidopsis* ATM also influences the status of γH2AX during meiosis is not known. We used immunolocalization to examine γH2AX foci in wild type and *atm* meiocytes. To avoid potential influences of ecotype difference in our cytological analyses, the *atm-5* mutant used was backcrossed with Col-0 wild type for four generations for cleaning its genetic background. Besides, we also introduced the Col-0 background *atm-2* mutant (Garcia et al., 2003) in our cytological analysis to further eliminate potential background affect. In wild type, γH2AX signals appeared as dots on leptotene chromosomes (Supplementary Figure S4A). The number of γH2AX foci increases in zygotene cells (204.07 ± 10.64, *n* = 30) (Figure 3A) and decreases at pachytene (80.20 ± 10.31, *n* = 30) (Figure 3B and Supplementary Figure S4B). However, in *atm-2* and *atm-5* mutants, while the rise and fall of γH2AX signals was similar to that in wild type (Figures 3A,B), the number of γH2AX foci in zygotene (*atm-2*: 146.84 ± 18.55, *n* = 32, *P* = 1.40E-11; *atm-5*: 152.30 ± 6.59, *n* = 30, *P* = 2.91E-11) and pachytene (*atm-2*: 47.31 ± 10.02, *n* = 31, *P* = 6.27E-11; *atm-5*: 51.07 ± 5.33, *n* = 30, *P* = 2.93E-11) meiocytes were less than those in wild type (204.07 ± 10.64, *n* = 30) (Figures 3C,D and Supplementary Table S3). These results demonstrate that ATM is required for normal levels of γH2AX during *Arabidopsis* meiosis.

In *Arabidopsis* mitotic cells, a second phosphatidylinositol 3-kinase like family member, Ataxia Telangiectasia and Rad3-related protein (ATR), is also involved in the formation of γH2AX (Friesner et al., 2005). To test whether the γH2AX foci in *atm* meiocytes are dependent on ATR, we examined γH2AX foci in *atm-5 atr-2* double mutant meiocytes. The *atm-5 atr-2* double mutant is completely sterile due to severe meiotic DSB repair defects, which is consistent with the previously reported phenotype of the *atm-1 atr-3* double mutant (Culligan and Britt, 2008). Our data showed that γH2AX foci are nearly abolished (20 ± 15.66, *n* = 65, *P* = 4.31E-11) in *atm-5 atr-2* early prophase I meiocytes (Figures 3A–D), indicating that the γH2AX foci remaining in *atm* mutants are ATR-dependent. In *atr-*2 single mutant meiocytes, the number of γH2AX foci in zygotene (208.48 ± 24.83, *n* = 33, *P* = 4.36E-01) and pachytene (72.44 ± 15.66, *n* = 32, *P* = 1.86E-02) (Figures 3A–D) are similar with those in wild type. Taken together, these data suggest that ATM is the primary phosphatidylinositol 3-kinase like family member responsible for meiotic γH2AX formation, whereas ATR acts as a back-up when ATM function is compromised.

### ATM Is Required for Normal Loading of RAD51 and DMC1 on Meiotic Chromosomes

**FIGURE 4:**
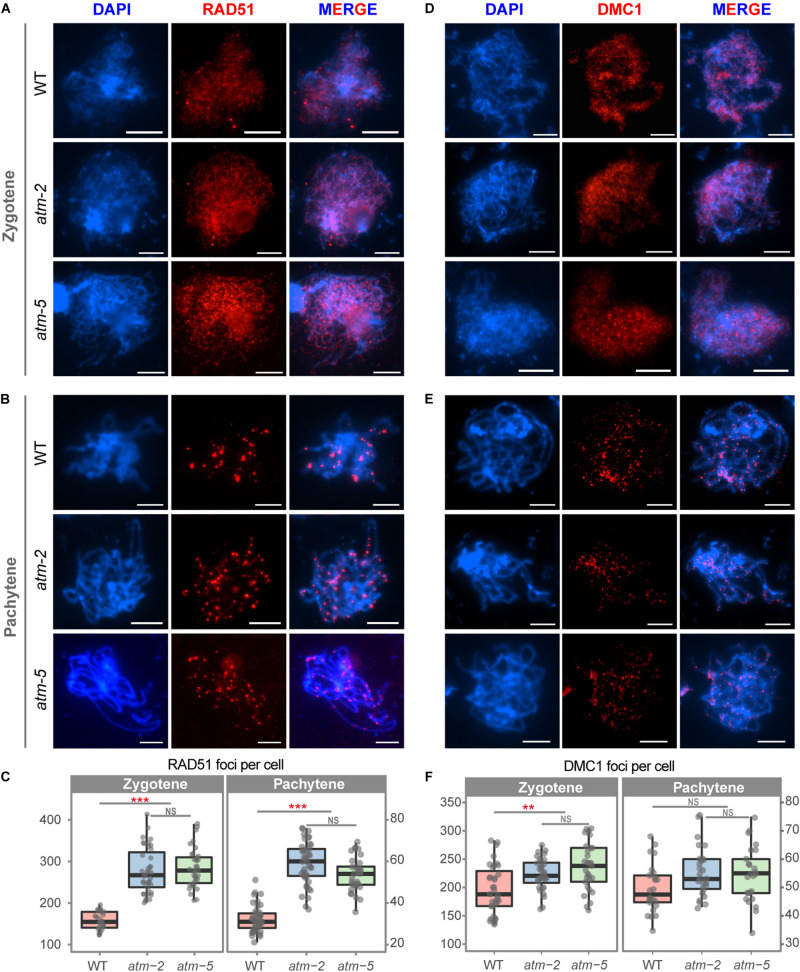
Immunolocalization of RAD51 and DMC1 in male meiocytes of wild type and *atm* mutants. **(A)** RAD51 localization on zygotene stage chromosomes of wild type, *atm-2* and *atm-5* respectively. **(B)** RAD51 localization in pachytene stage chromosomes of wild type, *atm-2* and *atm-5* respectively. **(C)** Number of RAD51 foci per cell of zygotene (WT: 168 ± 21.09, *n* = 27; *atm-2*: 279.15 ± 52.72, *n* = 40, *P* = 8.91E-12; *atm-5*: 285.67 ± 45.57, *n* = 39, *P* = 1.55E-11) and pachytene (WT: 32.15 ± 6.14, *n* = 47; *atm-2*: 58.84 ± 10.17, *n* = 43, *P* = 2.15E-15; *atm-5*: 53.6 ± 8.6, *n* = 32, *P* = 5.55E-13) stage cells in different alleles. **(D)** DMC1 localization on zygotene stage chromosomes of wild type, *atm-2* and *atm-5* respectively. **(E)** DMC1 localization in pachytene stage chromosomes of wild type, *atm-2* (and *atm-5* respectively. **(F)** Number of DMC1 foci per cell of zygotene (WT: 196.34 ± 49.39, *n* = 35; *atm-2*: 223.34 ± 28.02, *n* = 32, *P* = 3.38E-3; *atm-5*: 236.17 ± 42.17, *n* = 29, *P* = 4.97E-4) and pachytene (WT: 49.79 ± 8.61, *n* = 24; *atm-2*: 55.48 ± 8.77, *n* = 27, *P* = 3.35E-2; *atm-5*: 54.4 ± 9.76, *n* = 32, *P* = 6.24E-2) stage cells in different alleles. Bar = 5 μm. NS: *P* > 0.05 ***P < 0.001, **P < 0.01 (Wilcoxon Rank Sum test). Cells counted in each allele are from at least 30 plants. The *atm-2* and *atm-5* are two independent *atm* mutant alleles.)

The number of γH2AX foci may not be an accurate measure of the number of meiotic DSBs in *atm* because of phosphorylation defects. As an independent measure of the number of DSBs in *atm* meiocytes, we examined the immunolocalization of the recombinases RAD51 and DMC1 (Kurzbauer et al., 2012). Wild type zygotene meiocytes had an average of 168 (*SD* = 21.09, *n* = 27) RAD51 foci (Figures 4A,C), in agreement with former observations (Xue et al., 2018). However, we detected significantly more RAD51 foci at zygotene in *atm* meiocytes (*atm-2*: 279.15 ± 52.72, *n* = 40, *P* = 8.91E-12; *atm-5*: 285.67 ± 45.57, *n* = 39, *P* = 1.55E-11) (Figures 4A,C). Wild type zygotene meiocytes had an average of 196 DMC1 foci (*SD* = 49.39, *n* = 35), which increased to 223 DMC1 foci in *atm-2* (*SD* = 28.02, *n* = 32, *P* = 3.38E-03) and 236 DMC1 foci in *atm-5* (*SD* = 42.17, *n* = 29, *P* = 4.97E-04) (Figures 4D,F and Supplementary Tables S4, S5). Interestingly, RAD51 foci (70% increase) have a larger increase compared to DMC1 foci (20% increase).

Meiotic DSB repair defects often lead to persistent γH2AX, RAD51 and/or DMC1 foci on pachytene stage chromosomes (Li and Schimenti, 2007; Wang et al., 2012). Since we observed fewer γH2AX foci on *atm* pachytene chromosomes (Figure 3B) compared to wild type, we also analyzed RAD51 and DMC1 localization at pachytene in *atm*. The number of DMC1 pachytene foci in *atm-2* (55.48 ± 8.77, *n* = 27, *P* = 3.35E-02) and *atm-5* (54.40 ± 9.76, *n* = 25, *P* = 6.24E-02) are similar to wild type (49.79 ± 8.61, *n* = 24) (Figures 4E,F). However, the number of RAD51 pachytene foci in *atm-2* (58.84 ± 10.17, *n* = 43, *P* = 2.15E-15) and *atm-5* (53.60 ± 8.60, *n* = 32, *P* = 5.55E-13) are greater than wild type (32.15 ± 6.14, *n* = 47; Figures 4B,C). These results suggest that RAD51-dependent repair of meiotic DSBs is compromised in the absence of wild type ATM activity.

### There Are More Meiotic COs in *atm* Mutants

**FIGURE 5:**
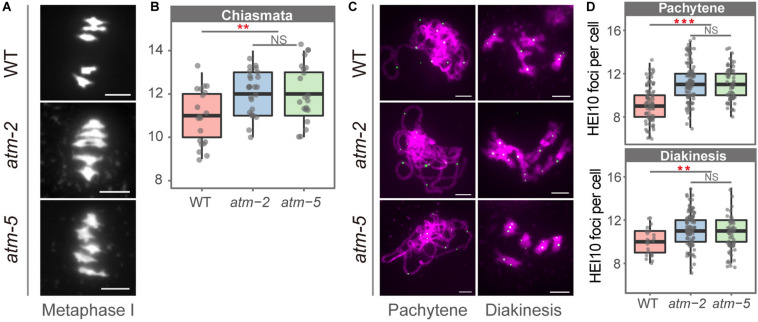
Number of chiasmata in wild type and *atm* mutants. **(A)** Bivalents in metaphase I meiocytes in wild type and *atm* mutants. The number of chiasmata of each chromosome was marked aside. **(B)** Number of chiasmata per cell in wild type (10.85 ± 1.18, *n* = 20) and *atm* mutants (*atm-2*: 12 ± 1.02, *n* = 25, *P* = 2.72E-3; *atm-5*: 12.01 ± 1.37, *n* = 21, *P* = 7.76E-3, Wilcoxon Rank Sum test). For each allele, metaphase I cells used for chiasmata counting are from about 30 plants. The *atm-2* and *atm-5* are two independent atm mutant alleles. **(C)** Immunolocalization of HEI10 (green dots) on pachytene (*atm-2*: 11.3 ± 1.82, *n* = 113, *P* = 7.59E-13; *atm-5*: 11.05 ± 1.47, *n* = 65, *P* = 3.25E-09) and diakinesis (*atm-2*: 11.25 ± 1.49, *n* = 110, *P* = 2.18E-04; *atm-5*: 10.94 ± 1.41, *n* = 48, *P* = 7.17E-03) stage chromosomes (magenta) in wild type and *atm* mutants. Bar = 5 μm. **(D)** Number of HEI10 foci in wild type and *atm* mutants. Cells counted in each allele are from at least 30 plants. The *atm-2* and *atm-5* are two independent *atm* mutant alleles.

To investigate whether ATM affects meiotic recombination, we compared the number of COs in *atm* and wild type meiocytes using cytological approach. We counted chiasmata, the cytological structures resulting from meiotic COs (Sanchez Moran et al., 2001), in metaphase I cells containing five bivalents from both wild type and *atm* mutants. The average number of chiasmata per meiocyte in wild type is 10.85 ± 1.18 (n = 20), which is in agreement with previous results (Osman et al., 2011). In the *atm* mutants the average number of chiasmata is significantly elevated (*atm-2*: 12.00 ± 1.02, *n* = 25, *P* = 2.72E-03; *atm-5*: 12.01 ± 1.37, *n* = 21, *P* = 7.76E-03) (Figures 5A,B and Supplementary Table S6).

To determine which type of COs are more abundant in *atm* mutants, we investigated the chromosome localization of HEI10, which is a Type I CO marker (Chelysheva et al., 2012), in pachytene and diakinesis stage meiocytes in both wild type and *atm* mutants (Figures 5C,D and Supplementary Table S8). In wild type we observed an average of 9.24 HEI10 foci (*SD* = 1.65, *n* = 86) per meiocytes at pachytene, and 9.96 (*SD* = 1.26, *n* = 23) at diakinesis (Figure 5D). In contrast, the average number of HEI10 foci per meiocyte in *atm* mutants is significantly higher on both pachytene (*atm-2*: 11.3 ± 1.82, *n* = 113, *P* = 7.59E-13; *atm-5*: 11.05 ± 1.47, *n* = 65, *P* = 3.25E-09) and diakinesis (*atm-2*: 11.25 ± 1.49, *n* = 110, *P* = 2.18E-04; *atm-5*: 10.94 ± 1.41, *n* = 48, *P* = 7.17E-03) stage chromosomes. These data suggest that an absence of ATM results in an elevation of Type I meiotic COs.

### Meiotic Chromosome Fragmentation Phenotypes Are Similar in *atm rad51* Double and *rad51* Single Mutants

**FIGURE 6:**
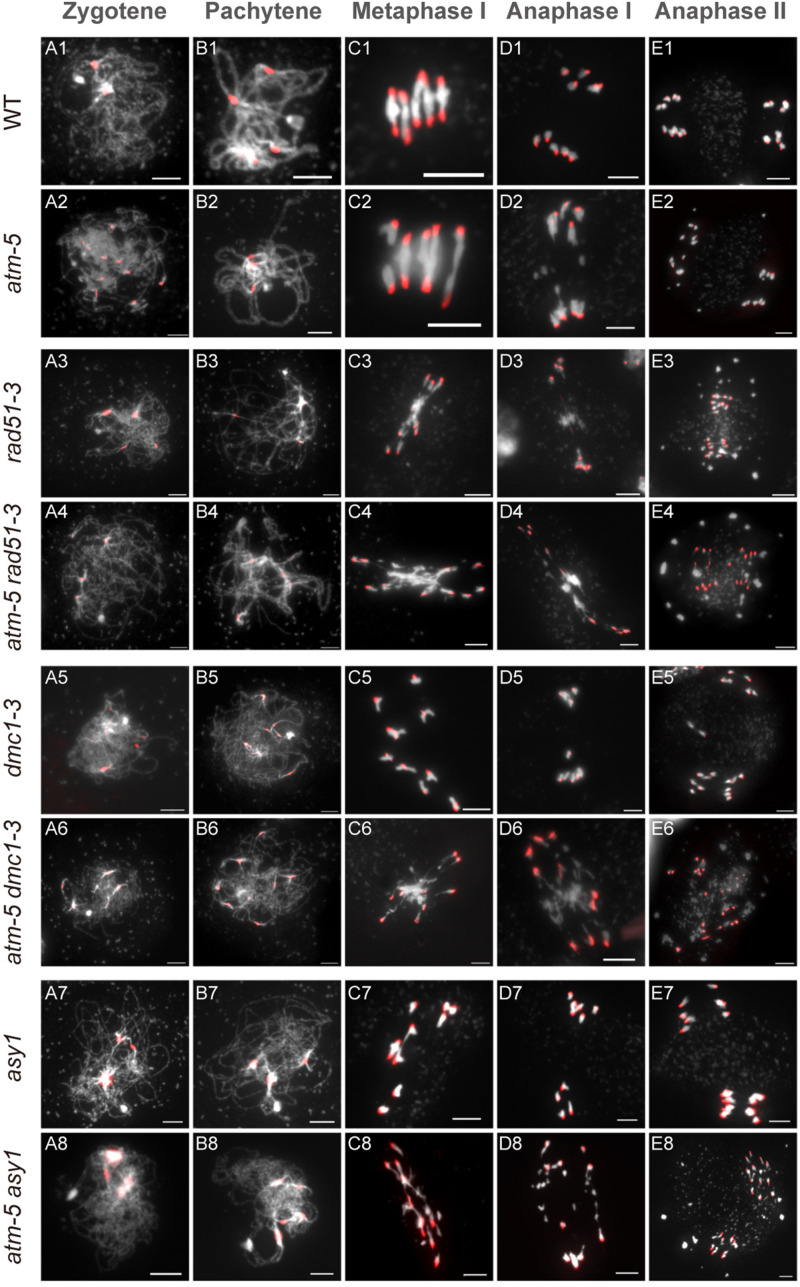
Chromosome morphology analysis of male meiocytes in different alleles. Wild type (WT) **(A1–E1)**, *atm-5* **(A2–E2)**, *rad51-3* **(A3–E3)**, *atm-5 rad51-3* **(A4–E4)**, *dmc1-3* **(A5–E5)**, *atm-5 dmc1-3* **(A6–E6)**, *asy1* **(A7–E7),** and *atm-5 asy1* **(A8–E8)** chromosome morphologies at zygotene, pachytene, metaphase I, anaphase I, and anaphase II. Centromere signals are in red. Bar = 5 μm.

The persistent RAD51 foci on *atm* pachytene chromosomes suggest that ATM influences RAD51 mediated DSB repair during meiosis. To explore the genetic relation between these two genes, we compared chromosome morphology and dynamics in *atm-5 rad51-3* double mutant with *atm-5*, *rad51-3*, and wild type. In wild type, homologous chromosomes start pairing and synapsis at zygotene (Figure 6A1). Synapsis is complete at pachytene, and chromosomes appear as thick threads (Figure 6B1). At metaphase I, five bivalents are well aligned on the equatorial plate (Figure 6C1). Zygotene and pachytene meiotic chromosomes in *atm-5* are indistinguishable from wild type (Figures 6A2,B2). We also examined the distribution of ASY1, ZYP1 and SYN1, which mediate axial element formation, central element formation, and sister chromatid cohesion, respectively (Cai et al., 2003; Higgins et al., 2005). We found that their distributions in *atm* mutants are indistinguishable from wild type (Supplementary Figure S3). However, at metaphase I, 29% of *atm-5* meiocytes have two early separated homologous chromosomes (Figures 6C2, 7A and Supplementary Table S7). Typical pachytene chromosomes were not observed in *rad51-3* (Figures 6A3,B3), which has severe chromosome fragmentation and entanglement phenotypes at metaphase I (Figure 6C3), consistent with previous observations (Li et al., 2004). In the *atm-5 rad51-3* double mutant, most prophase I meiocytes had leptotene or zygotene-like chromosome morphologies, but pachytene-like chromosomes were occasionally observed (9 of 193 prophase I cells) (Figure 6B4 and Supplementary Figure S5), suggesting improved synapsis of *rad51* chromosomes in the absence of ATM.

**FIGURE 7:**
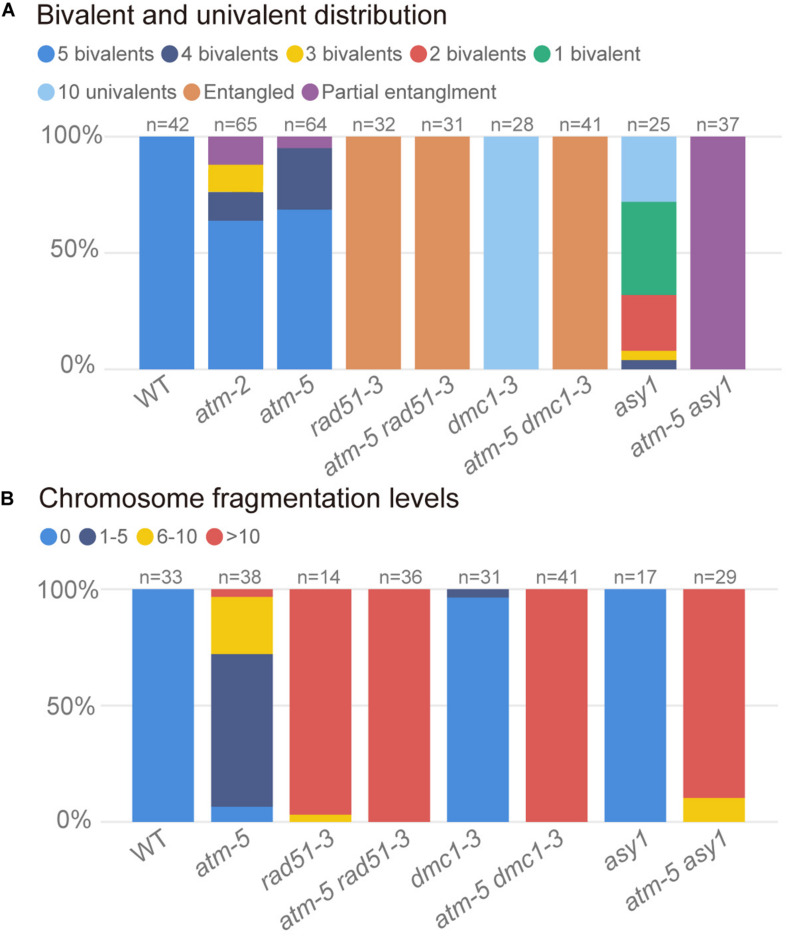
Quantification of bivalents and chromosome fragments in different alleles. **(A)** Quantification of bivalent and univalent in different alleles. The number of bivalents or univalents in each allele was counted in the metaphase I cells. For those metaphase I cells contain entangled chromosomes were classified as entangled. For those metaphase I cells contain both entangled chromosomes and univalents were classified as univalents and entangled. For each allele, the counted cells are from about 20 plants. **(B)** Quantification of chromosome fragmentation levels in different alleles. For each allele, the anaphase II and telophase II cells were observed after DAPI stained chromosome spreads and centromeric FISH. DAPI stained bodies without centromere signal were taken as chromosome fragments. Chromosome fragmentation levels are divided into four categories: 0 (no chromosome fragmentation), 1–5 (1–5 fragments per cell), 6–10 (6–10 fragments per cell) and >10 (more than 10 fragments per cell). For each allele, the counted cells are from about 12 plants.

In wild type, homologous chromosomes separate and migrate to opposite poles at anaphase I (Figure 6D1). Subsequently, sister chromatids separate at anaphase II, forming four nuclei with 5 chromosomes each (Figure 6E1). In contrast, at anaphase I and II, *atm-5* (Figures 6D2,E2), *rad51-3* (Figures 6D3,E3) and *atm-5 rad51-3* (Figures 6D4,E4) all have chromosome fragmentation phenotypes. Meiocytes from *atm-5* usually had 1–5 acentric fragments at anaphase II (Figure 7B), while *rad51-3* and *atm-5 rad51-3* always had more than 10 chromosome fragments (Figure 7B). The fact that *atm-5 rad51-3* has similar chromosome fragmentation levels compared to *rad51-3* implies that *ATM* and *RAD51* may be acting in the same meiotic DSB repair pathway in *Arabidopsis*.

### The *atm dmc1* Double Mutant Has More Severe Chromosome Fragmentation Than Either Single Mutant

Both RAD51 and the meiosis-specific DMC1 are homologs of bacterial RecA. To investigate the genetic relationship between ATM and DMC1, we compared the meiotic chromosome morphology phenotypes of *atm-5 dmc1-3* to wild type, *atm-5*, and *dmc1-3*. We found that unlike the typical pachytene chromosomes observed in wild type (Figure 6B1) and *atm-5* (Figure 6B2), both *dmc1-3* (104 prophase I meiocytes) and *atm-5 dmc1-3* (192 prophase I meiocytes) mutants had no detectable pachytene-like chromosomes (Figures 6B5,B6). At metaphase I, wild type meiocytes have five bivalents (Figure 6C1). However, *dmc1-3* has 10 univalents in all observed metaphase I meiocytes (*n* = 28, Figures 6C5, 7A). Interestingly, the *atm-5 dmc1-3* double mutant has entangled chromosomes (Figure 6C6), which were never observed in either *atm-5* or *dmc1-3* single mutant (Figures 6C2,C5). Homologous chromosomes segregate equally at anaphase I in wild type (Figure 6D1). In *dmc1-3*, univalents segregate randomly without any detectable chromosome fragmentations at anaphase I (Figure 6D5), indicating that meiotic DSBs have been repaired. However, in the double mutant, numerous chromosome fragments are present at anaphase I (Figure 6D6). At anaphase II, the chromosome fragmentation in *atm-5 dmc1-3* becomes more obvious after the separation of sister chromatids (Figure 6E6). Quantification of the number of chromosome fragments shows that the *atm-5 dmc1-3* double mutant has a more severe phenotype than the *atm-5* single mutant (Figure 6D2), but is similar to that of *rad51-3* and *atm-5 rad51-3* (Figure 7B). Taken together, the additive chromosome fragmentation phenotypes of *atm* and *dmc1* suggest that they act in different meiotic DNA repair pathways in *Arabidopsis*.

### The *atm asy1* Double Mutant Has More Chromosome Fragmentation Than Either Single Mutant

ASY1 is thought to be essential for DMC1-dependent inter-homolog recombination during meiosis. Loss of ASY1 eliminates the majority of inter-homolog recombination, resulting in repair of meiotic DSBs using sister chromatids as templates (Sanchez-Moran et al., 2007). We compared meiotic chromosome conformation phenotypes in an *atm-5 asy1* double mutant to wild type, *atm-5*, and *asy1*. Prophase I meiocytes from *asy1* (*n* = 74) and *atm-5 asy1* (*n* = 102) lack the typical pachytene chromosomes seen in wild type and *atm-5* (Figures 6B7,B8). At metaphase I, wild type meiocytes have five well aligned bivalents (Figure 6C1); and most *atm-5* meiocytes have four bivalents (Figures 6C2, 7A and Supplementary Figure S8). In *asy1*, most meiocytes have a mixture of univalents and bivalents in metaphase I (Figure 6C7), which is consistent with previous observations (Caryl et al., 2000). Interestingly, *atm-5 asy1* metaphase I meiocytes have univalents and entangled chromosomes (Figure 6C8), which is a more severe phenotype than either *atm-5* (Figure 6C2) or *asy1* (Figure 6C7) single mutants. These entanglements may be due to non-homolog interactions in the double mutant. Homologous chromosomes segregate unequally in *asy1* at anaphase I, but there is no detectable chromosome fragmentation (Figure 6D7). However, in the *atm-5 asy1* double mutant, numerous chromosome fragments were observed at anaphase I (Figure 6D8). The *atm-5 asy1* chromosome fragmentation phenotype is more obvious in anaphase II when sister chromatids separate (Figure 6E8). Quantification of the chromosome fragments showed the phenotype is more severe in *atm-5 asy1* compared *atm-5*, but slightly weaker than that seen in *rad51-3, atm-5 rad51-3*, or *atm-5 dmc1-3* (Figure 7B). Taken together, the additive chromosome fragmentation phenotypes of *atm* and *asy1* suggest that they work in different meiotic DNA repair pathways in *Arabidopsis*.

## Discussion

### ATM Is Potentially Involved in Meiotic DSBs Formation in *Arabidopsis*

To facilitate meiotic recombination, each meiocyte needs to generate sufficient DSBs at the onset of meiosis. *Arabidopsis* is thought to have about 200 DSBs per meiocyte (Choi et al., 2018). But the molecular mechanisms that control the number and distribution of meiotic DSBs are largely unknown. In yeast and mouse, ATM negatively regulates meiotic DSB levels (Cooper et al., 2014). *Arabidopsis* ATM also has meiotic functions but its influence on meiotic DSBs has not been reported prior to our study. We used immunolocalization of γH2AX as a proxy for meiotic DSBs numbers (Hunter et al., 2001) and showed that foci numbers were decreased in both *atm-2* and *atm-5* alleles (Figure 3). In mouse, ATM also mediates γH2AX foci formation (Widger et al., 2018). However, in mice ATM specifically contributes to the formation of γH2AX foci during leptotene (Widger et al., 2018). In *Arabidopsis*, ATM appears to influence γH2AX foci formation from leptotene to pachytene (Supplementary Figure S4A), suggesting differences in ATM’s meiotic function in different organisms. The mild decrease of γH2AX foci number in *atm* mutant and the elimination of γH2AX foci formation in *atm atr* double mutant (Figure 3) suggested that ATR is responsible for most γH2AX foci formation in *atm* mutant. Thus, it is intriguing to study how ATM and ATR choose to regulate different γH2AX foci formation during meiosis further. Studies in other species show that H2AX is directly phosphorylated by ATM during meiosis (Barchi et al., 2008; Cheng et al., 2013). Nonetheless, formal proof that *Arabidopsis* H2AX is a direct substrate of ATM will require more experimentation. In addition, it is possible that the decrease in γH2AX foci number in *atm* is due to unphosphorylated H2AX around DSBs, and does not reflect the true number of meiotic DSBs. Thus, it is rational to speculate that the repair of some DSBs without γH2AX localization might be affected in the absence of ATM. However, there is still lack of direct evidence that how γH2AX affect DSB repair. In *Arabidopsis*, γH2AX seems not required for mitotic DSB repair, as shown by the chromosome fusions observed in the *atr rad50* double mutant (Amiard et al., 2010, 2013). Meanwhile, mouse *h2ax* mutant displays male infertility due to unable to form sex body, but female *h2ax* mouse exhibit normal meiosis (Celeste et al., 2002). Thus, we speculate that it is more likely that the mild reduction of γH2AX in *Arabidopsis atm* mutant may not lead to meiotic DSB repair defects.

During meiosis, RAD51 and DMC1 binding to the processed single stranded overhangs and facilitating the strand invasion step of DSB repair (Kauppi et al., 2011; Pradillo et al., 2014; Widger et al., 2018). Immunolocalization of RAD51 and DMC1 showed that their foci numbers both increase during zygotene in *atm* mutants (Figure 4). Given that the conserved meiotic functions of ATM (Harper and Elledge, 2007; McKinnon, 2012), it is possible that *Arabidopsis atm* mutant also has elevated meiotic DSBs like the case in mouse and yeast *atm* mutants (Lange et al., 2011; Garcia et al., 2015). As a direct proxy of meiotic DSBs, the increased RAD51 and DMC1 foci in *atm* zygotene cells also implies that the absence of ATM elevates the number of meiotic DSBs in *Arabidopsis*. Although *Arabidopsis atm* mutant might have more meiotic DSBs, chromosome morphology analysis (Figure 1E) and ZYP1 localization analysis (Supplementary Figure S3) suggest that the synapsis is unaffected when ATM is absent. Besides, we did not observe any univalents in diakinesis cells in *atm*. Therefore, the increased number of COs in *atm* meiocytes without early separated homologous chromosomes (Figures 5A,B), which were used to measure CO numbers in this study, could properly reflect the meiotic recombination case in *atm*. Furthermore, our HEI10 immunolocalization analysis verifies the observation in CO number increasement and indicates that the increased COs in *atm* mutant are Type I COs (Figures 5C,D). This is consistent with the phenotypes observed in mouse (Barchi et al., 2008) rather than yeast *atm* mutants in which the increased COs are Type II COs (Anderson et al., 2015).

### ATM Plays an Important Role in Regulating Inter-Sister Repair of Meiotic DSBs

Meiocytes usually generate many more DSBs than COs. For example, in *Arabidopsis*, each meiocyte has over 200 DSBs (Choi et al., 2018), but only 9–10 COs (Lu et al., 2012). To maintain genome integrity, the remaining DSBs are thought to be repaired by the synthesis dependent strand annealing (SDSA) pathway to produce NCOs (McMahill et al., 2007). However, because NCO are difficult to detect unless they are accompanied by a gene conversion event, only a few have been documented in *Arabidopsis* (Lu et al., 2012; Sun et al., 2012; Wijnker et al., 2013). Furthermore, meiotic DSBs are believed to have a bias for using homologous chromosomes as repair templates (Lao and Hunter, 2010), but the strength of this bias has not be well characterized in plants. Therefore, it is possible that in *Arabidopsis* a significant number of meiotic DSBs might be repaired using sister chromatids as templates. Unfortunately, there is still no efficient way to directly detect sister-chromatid repair in flowering plants.

Here, we employed genetic studies to investigate functional relationships between the protein kinase ATM and other key meiotic factors, including DMC1, RAD51, and ASY1. In *dmc1* single mutants, COs are abolished and bivalents fail to form but there are no detectable chromosome fragments in meiosis (Figures 6, 7; Couteau et al., 1999; Sanchez-Moran et al., 2007), indicating that meiotic DSBs are completely repaired via RAD51-mediated IS repair. In contrast, we found that *atm dmc1* double mutants have a severe chromosome fragmentation phenotype not seen in *atm* or *dmc1* single mutants (Figures 6E6, 7B), suggesting that in the absence of ATM, inter-sister repair by RAD51 is impaired in the *dmc1* background. This is contrary to budding yeast, in which, ATM (Tel1) indirectly suppress RAD51 through a meiosis-specific kinase Mek1 (Carballo et al., 2008), thus its *dmc1* mutant exhibit severe meiotic DSB repair defects (Bishop et al., 1992). Considering that the *Arabidopsis dmc1* mutant does not have meiotic DSB repair defects and there was no evidence that *Arabidopsis* have a homolog of yeast Mek1. It is not surprising that *Arabidopsis* does not adopted a similar RAD51 suppression pathway. Meanwhile, our results suggest that ATM promoted RAD51-mediated IS repair in *Arabidopsis*. This idea is further supported by the observation that *atm asy1* double mutants have more severe meiotic chromosome fragmentation than *atm* single mutants (Figures 6, 7B). Since ASY1 is required for DMC1-mediated inter-homolog recombination (Sanchez-Moran et al., 2007), the double mutant is left with unrepaired breaks. In contrast, *atm rad51* double mutants have indistinguishable chromosome fragmentation levels compared to *rad51* single mutants (Figures 6, 7B), suggesting that ATM and RAD51 both function in IS meiotic repair.

We observed entangled chromosomes at metaphase I in both *atm dmc1* and *atm asy1* double mutants (Figures 6C6, 7A), which may be the result of ectopic non-homologous recombination. This aberrant phenotype is not observed in the corresponding single mutants, further supporting the idea that ATM functions in a parallel meiotic DSB repair pathway compared to DMC1 and ASY1. There are more RAD51 and DMC1 foci in *atm* meiocytes, but the increase in zygotene-stage RAD51 foci in *atm* meiocytes is much larger than that of DMC1 foci, indicating that ATM may affect the balance of RAD51 and DMC1 loading onto repair intermediates during meiosis. Moreover, we observed that *atm* pachytene meiocytes accumulate more RAD51 foci than that wild type, while DMC1 pachytene foci are at wild type levels (Figure 4). This suggests that RAD51-mediated DSB repair is delayed or impaired in *atm* mutants. Thus, we speculate that the DSB repair defects observed in *atm* mutants are due to impairment of the IS repair pathway.

**FIGURE 8:**
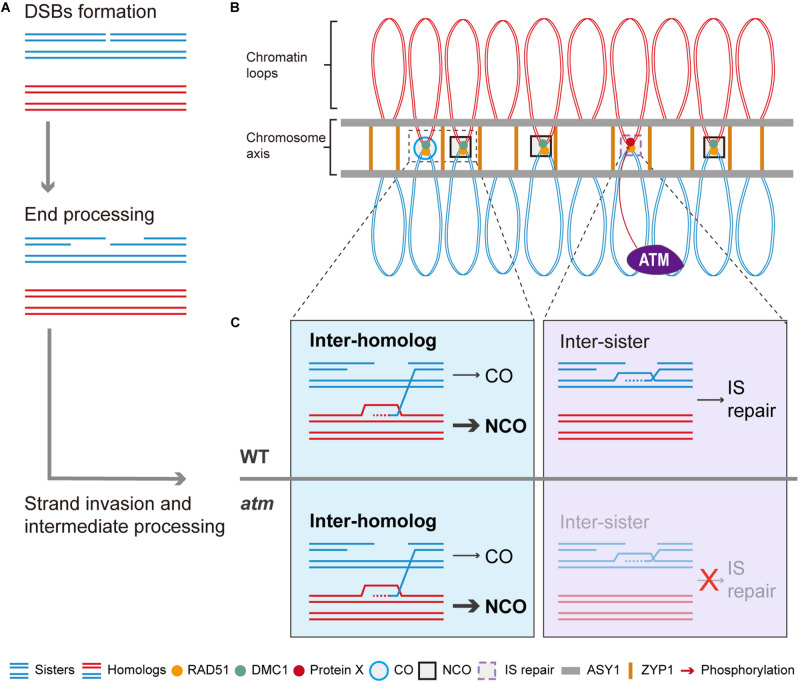
Model of ATM promote RAD51 mediated inter-sister meiotic DSB repair. After meiotic DSBs are formed, it will be processed to generated the 3’ ssDNAs **(A)**. With the help of RAD51 and DMC1, some of the ssDNAs will invade homologous chromosomes to seek template for repair **(A,B)**. Those DSBs repaired using homologous chromosomes will generate COs or NCOs while NCOs are in the majority **(B,C)**. Whereas some of the DSBs are repaired through inter-sister recombination which is mediated by RAD51 **(B,C)**. Thus, we proposed that ATM could promote this RAD51-mediated inter-sister repair process and it is possible that ATM regulate this process through phosphorylating a potential protein X **(C)**. Thus, when ATM is absent, this inter-sister repair pathway was impaired and leads to some unrepaired meiotic DSBs which eventually result in the mild chromosome fragmentations observed in *atm* mutants.

In summary, we propose a working model for ATM in meiotic recombination. In the model, after meiotic DSBs formation and resection (Figure 8A), the single strand DNA tails are coated with RAD51 and DMC1 and seek DNA templates to facilitate repair (Figure 8B). In wild type, those DSBs are repaired primarily by inter-homolog interactions, generating meiotic COs or NCOs (Figure 8C). However, even in wild type, some DSBs may be processed by RAD51-mediated inter-sister repair with the assistance of ATM (Figures 8B,C). In the absence of ATM, inter-homolog repair is still be mediated by DMC1 but IS repair is significantly compromised (Figure 8C), leading to unrepaired breaks and chromosome fragmentation. As ATM is an evolutionary and functionally conserved kinase (Xu et al., 1996; Garcia et al., 2003; Jones et al., 2012), its role in regulating IS repair may have relevance for other species including animals and fungi.

## Materials and Methods

### Plant Materials and Growth Conditions

The *atm-2* mutant used in this study were described in previous studies (Garcia et al., 2003; Waterworth et al., 2007). Another *atm* mutant is the newly identified *atm-5*. All the following quantitative analysis include these two independent *atm* mutants. Other mutant alleles used in this study are *spo11-1-1* (Grelon et al., 2001), *rad51-3* (Wang et al., 2012), *asy1* (Sanchez-Moran et al., 2007), *hei10-2* (Chelysheva et al., 2012), and *atr-2* (Culligan et al., 2004). The *dmc1-3* allele was a previously identified Ds insertion mutant in our laboratory. The Ds insertion is located in the first intron of *DMC1* (Supplementary Figure S6A). This expression of *DMC1* in *dmc1-3* is undetectable using primers across the insertion site (Supplementary Figure S6B). The *atm-5 atr-2*, *atm-5 dmc1-3*, and *atm-5 rad51-3* double mutants were generated by crossing the corresponding heterozygous mutants with heterozygous *ATM*/*atm-5* plants. All materials were grown at 20–22°C under long day conditions (16 h light/8 h dark). Primers used for genotyping are listed in Supplementary Table S1.

### Characterization of Mutant Phenotypes

Pollen viability was analyzed through Alexander staining on pre-dehiscent anthers according to a simplified protocol (Peterson et al., 2010). For tetrad morphology analysis, tetrad-stage male meiocytes were squeezed from fresh anthers onto glass microscope slides and stained with Toluidine Blue solution (0.025%, m/v). After covering with cover glass, the stained tetrads were examined using an Axio Imager A2 microscope (Zeiss, Heidelberg, Germany) under white light illumination.

### Map-Based Cloning and Mutation Identification

To construct the mapping population, we crossed the heterozygous *atm-5* (L*er* background) to wild type Col-0. An F2 population was generated by allowing F1 plants to self-fertilize. From 586 F2 plants, 143 sterile plants were isolated and used for mapping. Genetic markers for map-based cloning of the *atm-5* mutation were designed based on simple sequence length polymorphisms (SSLP) between Columbia (Col-0) and Landsberg *erecta* (L*er*). Primers for amplifying SSLPs are listed in Supplementary Table S2. The entire *ATM* gene was divided to seven overlapping segments for cloning by PCR. Each segment was cloned into the pEASY-T1 vector (CT111-02, Transgen Biotech, Beijing, China) and a single colony containing for each segment was selected for sequencing. The full cDNA of *ATM* was also cloned using a similar approach with six segments into the cloning vector for sequencing. Primers sequences are listed in Supplementary Table S1.

### Expression Analysis

Semi-quantitative RT-PCR was used to analyze the expression of *ATM* in different *atm* mutant alleles. Total RNA from wild type and mutant inflorescences was isolated using the Trizol reagent (Cat^#^: 15596018, Thermo Fisher Scientific). The first strand of cDNA was synthesized using the PrimeScript 1st strand cDNA synthesis kit (Cat^#^: 6110B, Takara, Beijing, China). Semi-quantitative RT-PCR was conducted with the 2 × Taq master mix (Cat^#^: E005-01B, Novoprotein, Shanghai, China). Primers sequences are listed in Supplementary Table S1.

### Chromosome Spread and Immunolocalization Analyses

Chromosome spreads and centromere FISH were conducted as described previously (Wang et al., 2014). Immunolocalization experiments were performed following the previous description (Armstrong et al., 2009) with minor modifications. Briefly, after chromosome spreads were prepared, the slides were washed in wash buffer I (PBS, 1% Triton X-100, pH 7.4) for 1 h. Then these slides were blocked at 37°C for 1 h with blocking buffer (PBS, 5% BSA, 1 mM EDTA, pH 7.4). The primary antibody was diluted to working concentration with blocking buffer and then added to the slides. The slides were covered with parafilm and incubated overnight at 4°C in a moisture chamber. After incubation, these slides were washed with washing buffer II (PBS, 0.1% Tween 20, pH 7.4) three times (15 min each time). The secondary antibody was diluted to working concentration with blocking buffer and then added to the slides. After covering with parafilm, the slides were incubated at 37°C in a moisture chamber in dark. The slides were then washed with washing buffer II three times (15 min each time) and mounted in 1.5 μg/mL DAPI (Vector Laboratories, Burlingame, CA, United States). The working concentration of primary antibodies are γH2AX at 1/200 dilution, DMC1 at 1/800 dilution, RAD51 at 1/50 dilution, ZYP1 at 1/50 dilution and ASY1 at 1/200 dilution. Immunolocalization of SYN1 (1/200 dilution), HEI10 (1/400 dilution) and ASY1 (1/200 dilution) was carried out as described previously (Chelysheva et al., 2013). Rabbit polyclonal RAD51, ASY1, SYN1 and rat polyclonal ZYP1 antibodies were generated and verified previously (Wang et al., 2012, 2016; Niu et al., 2015). Rabbit polyclonal DMC1 antibody was generated as described previously (Wang et al., 2019). Rabbit polyclonal γH2AX antibody was generated and tested previously (Zhou et al., 2016). The HEI10 polyclonal antibody was generated through immune rabbit with a KLH conjugated peptide sequence of HEI10 (PKDEIWPARQNS). The γH2AX, HEI10 and DMC1 antibody was further verified through immunolocalization in *spo11-1-1*, *dmc1-3*, *hei10-2* respectively (Supplementary Figure S7). Secondary antibodies used in this study were Alexa Fluor 488 Goat Anti-Mouse IgG (H + L) (A32723, 1/1000 dilution), Alexa Fluor 555 Goat Anti-Rabbit IgG (H + L) (A32732, 1/1000 dilution) and Alexa Fluor 488 Goat Anti-Rabbit IgG (H + L) (A32731, 1/1000 dilution) (Invitrogen, Carlabad, CA, United States). Microscopy was carried out using an Axio Imager A2 microscope (Zeiss, Heidelberg, Germany). Images were processed with Image J (Collins, 2007) and figures were constructed using Photoshop CS3 and Illustrator CS6 (Adobe, Mountain View, CA, United States). To count the number of fluorescent foci signals, images of chromosome signal and protein foci signal were merged together without any processing. Only those foci signals merged onto chromosome signal were counted through the Image J count tool. The cells used for counting γH2AX foci, RAD51 foci, DMC1 foci or HEI10 foci in each allele are from at least 6 slides. Each slide contains young flower buds from about 5 plants. The cells used for chiasmata counting are from about 15 slides and each slide contains samples from two plants. The cells used for bivalent counting are from about 10 slides and each slide contain samples from two plants. The cells used for chromosome fragments counting are from about 6 slides and each slide contains samples from two plants. The quantitative analysis of γH2AX, RAD51, DMC1 foci, HEI10 foci number and chiasmata number in *atm* mutant both include two independent mutant alleles (*atm-2* and *atm-5*). The qualitative analysis of γH2AX foci localization and meiotic chromosome morphology all include one representative single or double mutant allele.

### Statistical Methods

Statistical comparisons of the γH2AX foci, RAD51 foci, DMC1 foci, HEI10 foci and chiasmata number was carried out in R using the Wilcoxon Rank Sum test.

## Data Availability Statement

The raw data supporting the conclusions of this article will be made available by the authors, without undue reservation, to any qualified researcher.

## Author Contributions

PL and YY designed the research. YY, WC, HL, LM, DR, and AM performed the genetic mapping experiments and molecular analysis. YY performed the molecular and cytological analyses. YY, XL, and PL analyzed the data. YY wrote a draft for the manuscript. PL revised the draft and wrote the manuscript. All authors read and approved the final manuscript.

## Conflict of Interest

The authors declare that the research was conducted in the absence of any commercial or financial relationships that could be construed as a potential conflict of interest.

## Supplementary Material

FIGURE S1 Confirmation of the mutated gene in line 184 was *ATM*. **(A)** Sequence of the identified 766 bp insertion in *ATM* gene of *line 184* mutant. **(B)** Blast results of this 766 bp segment against the *Arabidopsis* genome sequence. In each chromosome, there exist a locus that is highly similar with this segment. **(C)** Male meiosis chromosome morphology at anaphase I of *atm-2*/*atm-2* and *line 184*/*atm-2* (*n* = 2). Bar = 5 μm.

FIGURE S2 Part of the *ATM* cDNA sequencing results from *atm-5*. **(A)** Alignment of partial *ATM* CDS sequencing results in *atm-5* with wild type *ATM* CDS. NM_001339356.1, NM_001229355.1 and NM_001339354.1 are annotated transcripts of *ATM* gene in NCBI. While, the 1, 2, 3, and 4 are different transcriptional products of *ATM* in *atm-5* mutant around the insertion site. **(B)** A sketch map of putative truncated ATM protein product in *atm-5* mutant.

FIGURE S3 Immunolocalization of AYS1, ZYP1 and SYN1 in male meiocytes of *atm* mutants and wild type. **(A)** Zygotene stage chromosomes (blue) with ASY1 signal (red) and ZYP1 signal (green). **(B)** Pachytene stage chromosomes (blue) with ASY1 signal (red) and ZYP1 signal (green). **(C)** Chromosomes with SYN1 (red) signal in wild type and *atm* mutants at different meiotic stages. Chromosomes were stained with DAPI (blue). In each line, at least 20 meiocytes were examined. Bar = 5 μm. The *atm-2* and *atm-5* are two independent atm mutant alleles.

FIGURE S4 γH2AX localization in leptotene and pachytene meiocytes of wild type and *atm* mutants. **(A)** Immunolocalization of γH2AX in leptotene meiocytes of wild type and *atm* mutants. **(B)** Dual-immunolocalization of γH2AX (red) and ZYP1 (green) in pachytene meiocytes of wild type and *atm* mutants. Chromosomes were stained with DAPI (blue). Bar = 5μm. The *atm-2* and atm-5 are two independent atm mutant alleles.

FIGURE S5 The observation of near fully synapsed chromosomes in *atm-5 rad51-3* double mutant. DAPI stained pachytene-like chromosome spreads of *rad51-3* and *atm-5 rad51-3.* Bar = 5 μm.

FIGURE S6 Characterization of *dmc1-3* mutant used in this study. **(A)** The exon and intron structure of *DMC1* gene in Arabidopsis. The Ds insertion in *dmc1-3* mutant was identified by PCR through Ds specific primers. The position of Ds insertion is indicated in the picture. **(B)** RT-PCR analysis of DMC1 expression in *dmc1-3* mutant and wild type. No DMC1 mRNA is produced across the Ds insertion site in *dmc1-3* mutant. The positions of primers are indicated in **(A)**.

FIGURE S7 Verification of antibodies used in this study through immunolocalization against wild type plants and corresponding mutants. **(A)** Immunolocalization of DMC1 in zygotene meiocytes of wild type and *dmc1-3* mutant. **(B)** Immunolocalization of γH2AX in zygotene of wild type and *spo11-1-1* mutant. **(C)** Immunolocalization of HEI10 in diakinesis of wild type and *hei10-2* mutant.

FIGURE S8 Examples of metaphase I chromosome morphology for Figure 7A. Typical chromosome morphology of each type of metaphase I cells in different mutants exhibited in Figure 7A.

TABLE S1 List of primers used in this study.

TABLE S2 List of SSLP markers primers used in this study.

TABLE S3 Numbers of counted γH2AX signal foci in each image.

TABLE S4 Numbers of counted DMC1 immunolocalization signal foci in each image.

TABLE S5 Numbers of counted RAD51 immunolocalization signal foci in each image.

TABLE S6 Numbers of counted chiasmata in each image.

TABLE S7 Number of cells counted in each type of mutants.

TABLE S8 Number of counted HEI10 immunolocalization signal foci in each image.
