# Supplementary material for: ATM Promotes RAD51-Mediated Meiotic DSB Repair by Inter-Sister-Chromatid Recombination in Arabidopsis
Source: Front Plant Sci. 2020 Jun 25;11:839. doi: 10.3389/fpls.2020.00839 (PMC7329986; doi:10.3389/fpls.2020.00839)
Supplement: FIGURE S1 — Confirmation of the mutated gene in line 184 was ATM. (A) Sequence of the identified 766 bp insertion in ATM gene of line 184 mutant. (B) Blast results of this 766 bp segment against the Arabidopsis genome sequence. In each chromosome, there exist a locus that is highly similar with this segment. (C) Male meiosis chromosome morphology at anaphase I of atm-2/atm-2 and line 184/atm-2 (n = 2). Bar = 5 μm. [file Data_Sheet_1.PDF]

Figure. S1

A

48th intron

```
······tttgttgttaacagCAATGTTTTTAAAACCGACCCACACAGTGAACCGGACTACCTTCTGGGTCACCGGGTCATCGGGTCAACCGGGTTGAA
CCACGGGCCAATAAATTAAGTAATAAATTTAATATTAATAGAGATTTTATAGTTTTTATAAATAGAAAACAAAATAATCATATCATGTTTAATAGAAA
ACAAAATAATCATATCATGTTTAATAAAAAATATAAAATCATATTATGTTTGATAAAATATAAAATCATTTACGCCAAAAAAACATATATATATATAT
ATATATATATAAAATCAAACAATAATAAGAAAGTGATTAAAAAATTTAAATTCAACTAAATGTCTAACATCGTAACAAATATAATAGAAGATCACCAA
TGATAGAAACAAACTAATGAAACATCGTGAATTCAAACAACTAATCTCGTTTGCATATATCACCATTCGATTCATTTTTAAGGCCGCATTGTGAAATC
TTCACCAAAATCTAAAACATCAAATGAATTTAAGAACTAAAAAAACTTATAAAAATAATTGAAGAAAGTTTAAAACCCCACATTTTATTCCAAAATGA
AGAGAATTTTTTAAAACCCACATCCGGCTGGTTCAACCGATTCACCGGGTCATGGGTTAATGGCGAGTTTTTGTGGGTTTTTTCGGTTTTGAATTTGT
CGGGTTTTAGCATTAAACCCAAACCGGAAAAGTGTTCAGGTCACGGGTTAACCGGTCGGACCGGGCGGTTCGGGTCGGGCGTGAAAACACTGGTTAAC
AGttaccggatcatgttgagatacttgtgtctgcaat······
```

49th exon

B

Description	Max score	Total score	Query cover	E value	Ident	Accession
Arabidopsis thaliana chromosome 3 sequence	1072	4815	100%	0.0	93%	NC_003074.8
Arabidopsis thaliana chromosome 1 sequence	1044	3283	99%	0.0	96%	NC_003070.9
Arabidopsis thaliana chromosome 5 sequence	811	4157	99%	0.0	86%	NC_003076.8
Arabidopsis thaliana chromosome 2 sequence	686	1250	98%	0.0	84%	NC_003071.7
Arabidopsis thaliana chromosome 4 sequence	392	458	79%	6e-108	79%	NC_003075.7

C

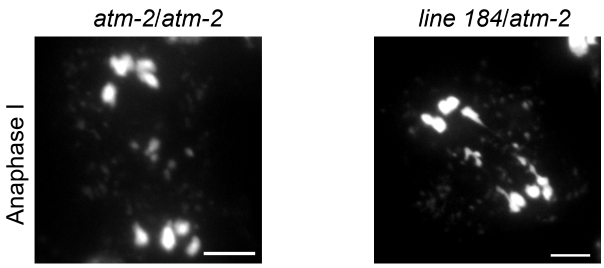

Figure. S1 Confirmation of the mutated gene in line 184 was *ATM*.
(A) Sequence of the identified 766 bp insertion in *ATM* gene of *line 184* mutant. **(B)** Blast results of this 766 bp segment against the *Arabidopsis* genome sequence. In each chromosome, there exist a locus that is highly similar with this segment. **(C)** Male meiosis chromosome morphology at anaphase I of *atm-2*/*atm-2* and *line 184*/*atm-2* (n = 2). Bar = 5 μm.
