# Supplementary material for: ATM Promotes RAD51-Mediated Meiotic DSB Repair by Inter-Sister-Chromatid Recombination in Arabidopsis
Source: Front Plant Sci. 2020 Jun 25;11:839. doi: 10.3389/fpls.2020.00839 (PMC7329986; doi:10.3389/fpls.2020.00839)
Supplement: FIGURE S2 — Part of the ATM cDNA sequencing results from atm-5. (A) Alignment of partial ATM CDS sequencing results in atm-5 with wild type ATM CDS. NM_001339356.1, NM_001229355.1 and NM_001339354.1 are annotated transcripts of ATM gene in NCBI. While, the 1, 2, 3, and 4 are different transcriptional products of ATM in atm-5 mutant around the insertion site. (B) A sketch map of putative truncated ATM protein product in atm-5 mutant. [file Data_Sheet_2.PDF]

Figure. S2

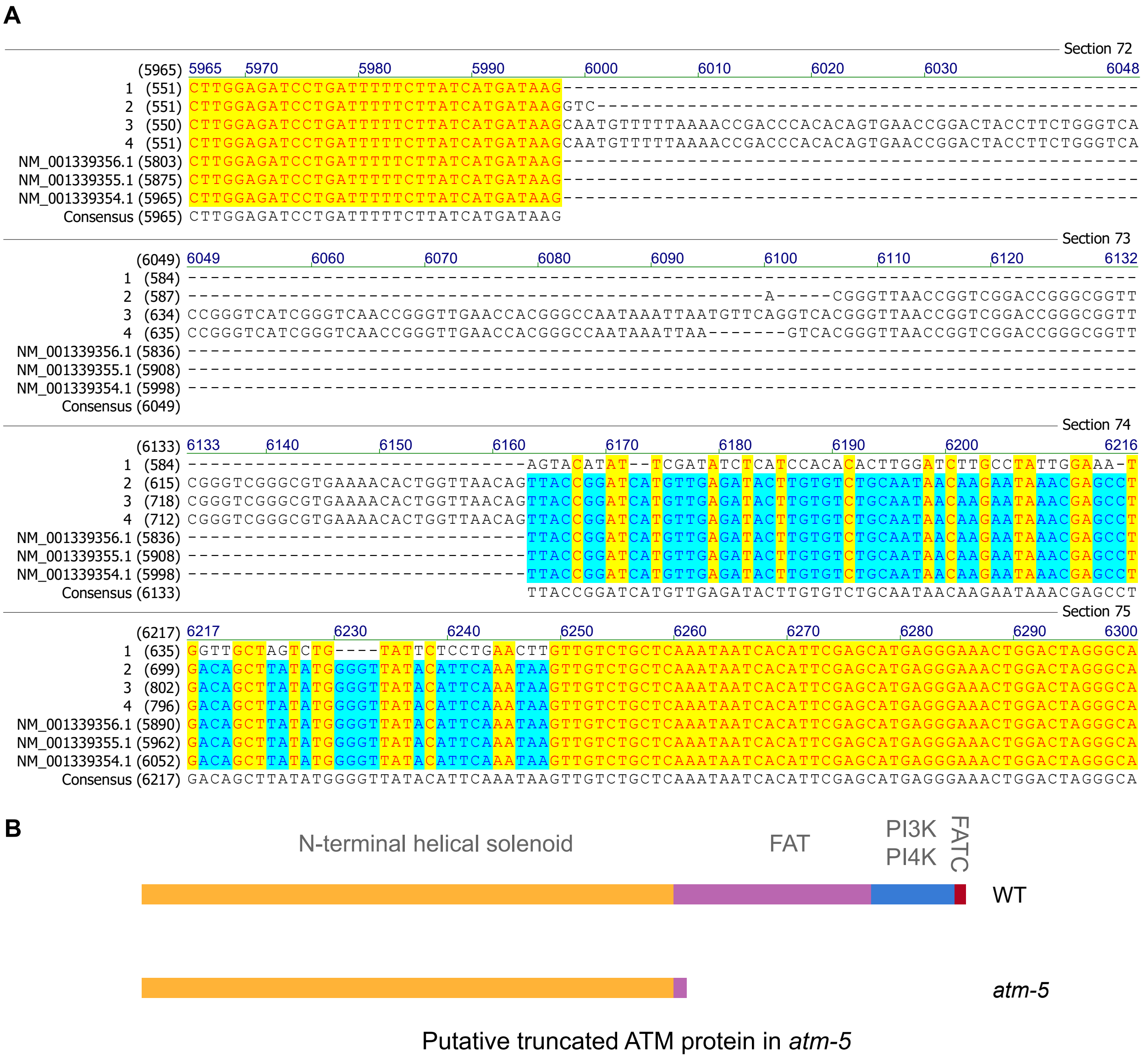

Figure. S2 Part of the *ATM* cDNA sequencing results from *atm-5*.

(A) Alignment of partial *ATM* CDS sequencing results in *atm-5* with wild type *ATM* CDS. NM_001339356.1, NM_001229355.1 and NM_001339354.1 are annotated transcripts of *ATM* gene in NCBI. While, the 1, 2, 3 and 4 are different transcriptional products of *ATM* in *atm-5* mutant around the insertion site. **(B)** A sketch map of putative truncated ATM protein product in *atm-5* mutant.
