# Supplementary material for: ATM Promotes RAD51-Mediated Meiotic DSB Repair by Inter-Sister-Chromatid Recombination in Arabidopsis
Source: Front Plant Sci. 2020 Jun 25;11:839. doi: 10.3389/fpls.2020.00839 (PMC7329986; doi:10.3389/fpls.2020.00839)
Supplement: FIGURE S3 — Immunolocalization of AYS1, ZYP1 and SYN1 in male meiocytes of atm mutants and wild type. (A) Zygotene stage chromosomes (blue) with ASY1 signal (red) and ZYP1 signal (green). (B) Pachytene stage chromosomes (blue) with ASY1 signal (red) and ZYP1 signal (green). (C) Chromosomes with SYN1 (red) signal in wild type and atm mutants at different meiotic stages. Chromosomes were stained with DAPI (blue). In each line, at least 20 meiocytes were examined. Bar = 5 μm. The atm-2 and atm-5 are two independent atm mutant alleles. [file Data_Sheet_3.PDF]

Figure. S3

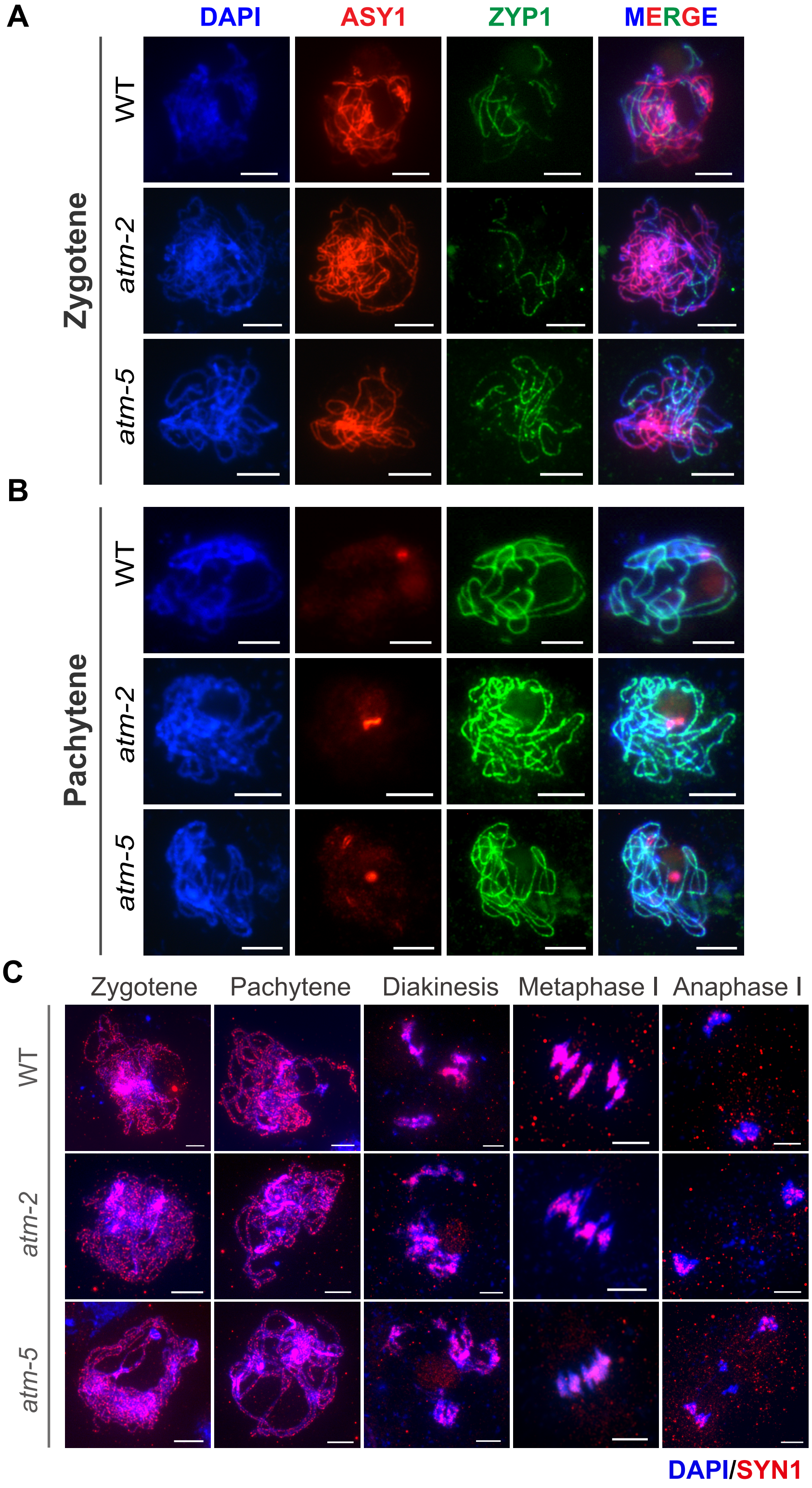

Figure. S3 Immunolocalization of AYS1, ZYP1 and SYN1 in male meiocytes of *atm* mutants and wild type.

(A) Zygotene stage chromosomes (blue) with ASY1 signal (red) and ZYP1 signal (green). **(B)** Pachytene stage chromosomes (blue) with ASY1 signal (red) and ZYP1 signal (green). **(C)** Chromosomes with SYN1 (red) signal in wild type and *atm* mutants at different meiotic stages. Chromosomes were stained with DAPI (blue). In each line, at least 20 meiocytes were examined. Bar = 5 μm.
