# Supplementary material for: ATM Promotes RAD51-Mediated Meiotic DSB Repair by Inter-Sister-Chromatid Recombination in Arabidopsis
Source: Front Plant Sci. 2020 Jun 25;11:839. doi: 10.3389/fpls.2020.00839 (PMC7329986; doi:10.3389/fpls.2020.00839)
Supplement: FIGURE S4 — γH2AX localization in leptotene and pachytene meiocytes of wild type and atm mutants. (A) Immunolocalization of γH2AX in leptotene meiocytes of wild type and atm mutants. (B) Dual-immunolocalization of γH2AX (red) and ZYP1 (green) in pachytene meiocytes of wild type and atm mutants. Chromosomes were stained with DAPI (blue). Bar = 5μm. The atm-2 and atm-5 are two independent atm mutant alleles. [file Data_Sheet_4.PDF]

Figure. S4

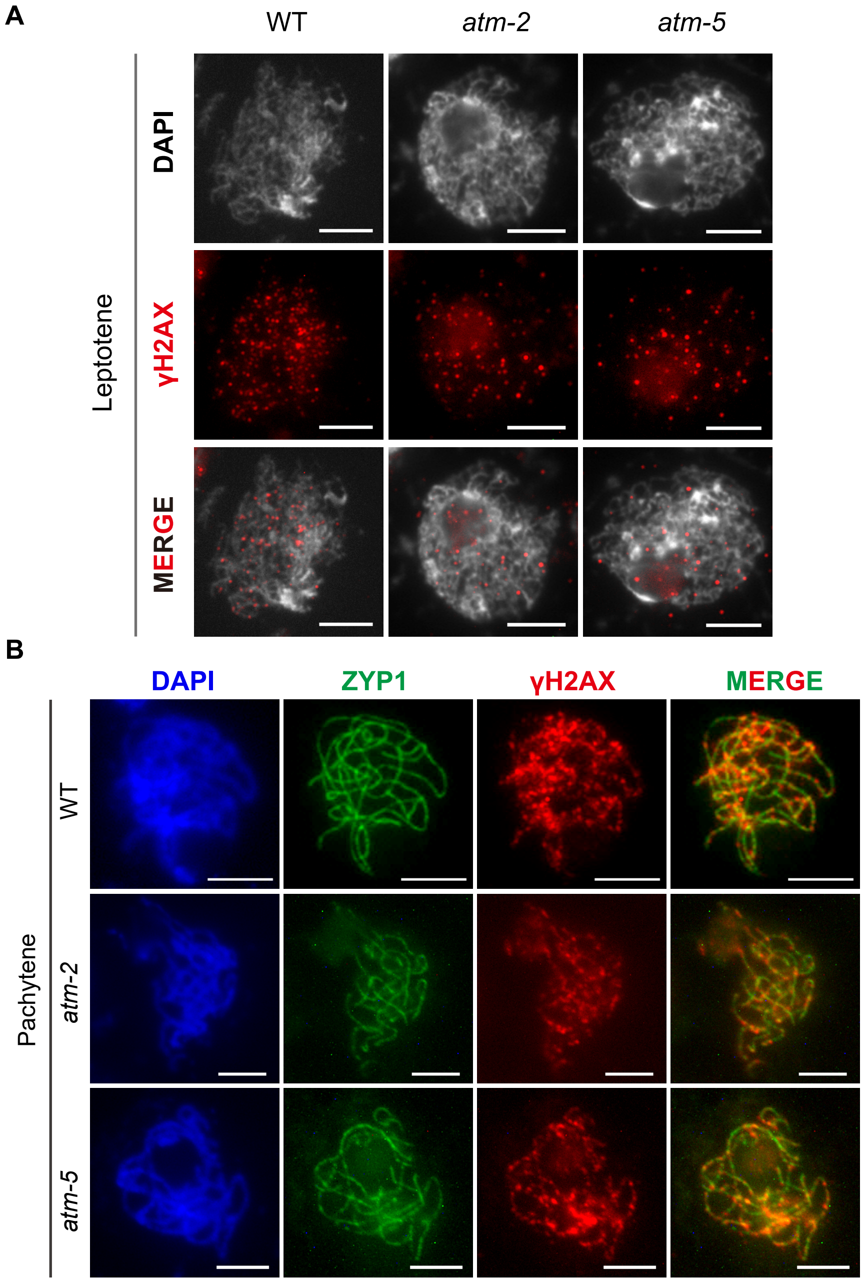

Figure. S4 γH2AX localization in leptotene and pachytene meiocytes of wild type and *atm* mutants.

(A) Immunolocalization of γH2AX in leptotene meiocytes of wild type and *atm* mutants. **(B)** Dual-immunolocalization of γH2AX (red) and ZYP1 (green) in pachytene meiocytes of wild type and *atm* mutants. Chromosomes were stained with DAPI (blue). Bar = 5μm.
