# Supplementary material for: ATM Promotes RAD51-Mediated Meiotic DSB Repair by Inter-Sister-Chromatid Recombination in Arabidopsis
Source: Front Plant Sci. 2020 Jun 25;11:839. doi: 10.3389/fpls.2020.00839 (PMC7329986; doi:10.3389/fpls.2020.00839)
Supplement: FIGURE S5 — The observation of near fully synapsed chromosomes in atm-5 rad51-3 double mutant. DAPI stained pachytene-like chromosome spreads of rad51-3 and atm-5 rad51-3. Bar = 5 μm. [file Data_Sheet_5.PDF]

Figure. S5

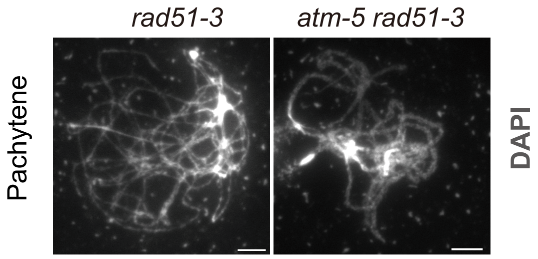

Figure. S5 The observation of near fully synapsed chromosomes in *atm-5 rad51-3* double mutant.

DAPI stained pachytene-like chromosome spreads of *rad51-3* and *atm-5 rad51-3.* Bar = 5 μm.
