# Supplementary material for: ATM Promotes RAD51-Mediated Meiotic DSB Repair by Inter-Sister-Chromatid Recombination in Arabidopsis
Source: Front Plant Sci. 2020 Jun 25;11:839. doi: 10.3389/fpls.2020.00839 (PMC7329986; doi:10.3389/fpls.2020.00839)
Supplement: FIGURE S6 — Characterization of dmc1-3 mutant used in this study. (A) The exon and intron structure of DMC1 gene in Arabidopsis. The Ds insertion in dmc1-3 mutant was identified by PCR through Ds specific primers. The position of Ds insertion is indicated in the picture. (B) RT-PCR analysis of DMC1 expression in dmc1-3 mutant and wild type. No DMC1 mRNA is produced across the Ds insertion site in dmc1-3 mutant. The positions of primers are indicated in (A). [file Data_Sheet_6.PDF]

## Figure. S6

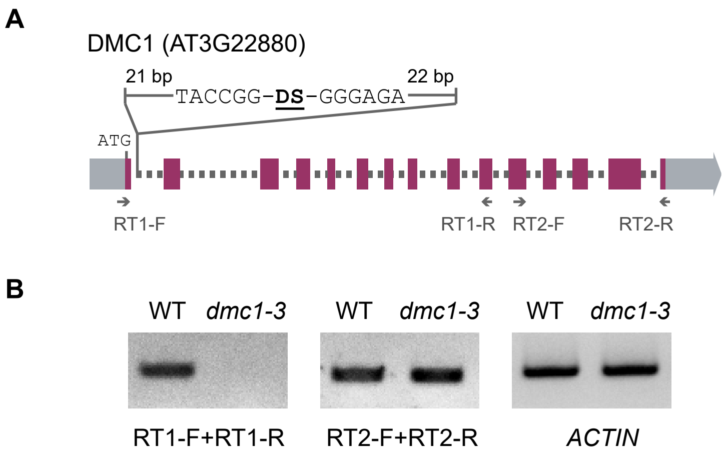


**Figure. S6 Characterization of *dmc1-3* mutant used in this study.**
**(A)** The exon and intron structure of *DMC1* gene in Arabidopsis. The Ds insertion in *dmc1-3* mutant was identified by PCR through Ds specific primers. The position of Ds insertion is indicated in the picture. **(B)** RT-PCR analysis of DMC1 expression in *dmc1-3* mutant and wild type. No DMC1 mRNA is produced across the Ds insertion site in *dmc1-3* mutant. The positions of primers are indicated in **(A)**.
