# Supplementary material for: ATM Promotes RAD51-Mediated Meiotic DSB Repair by Inter-Sister-Chromatid Recombination in Arabidopsis
Source: Front Plant Sci. 2020 Jun 25;11:839. doi: 10.3389/fpls.2020.00839 (PMC7329986; doi:10.3389/fpls.2020.00839)
Supplement: FIGURE S7 — Verification of antibodies used in this study through immunolocalization against wild type plants and corresponding mutants. (A) Immunolocalization of DMC1 in zygotene meiocytes of wild type and dmc1-3 mutant. (B) Immunolocalization of γH2AX in zygotene of wild type and spo11-1-1 mutant. (C) Immunolocalization of HEI10 in diakinesis of wild type and hei10-2 mutant. [file Data_Sheet_7.PDF]

Figure. S7

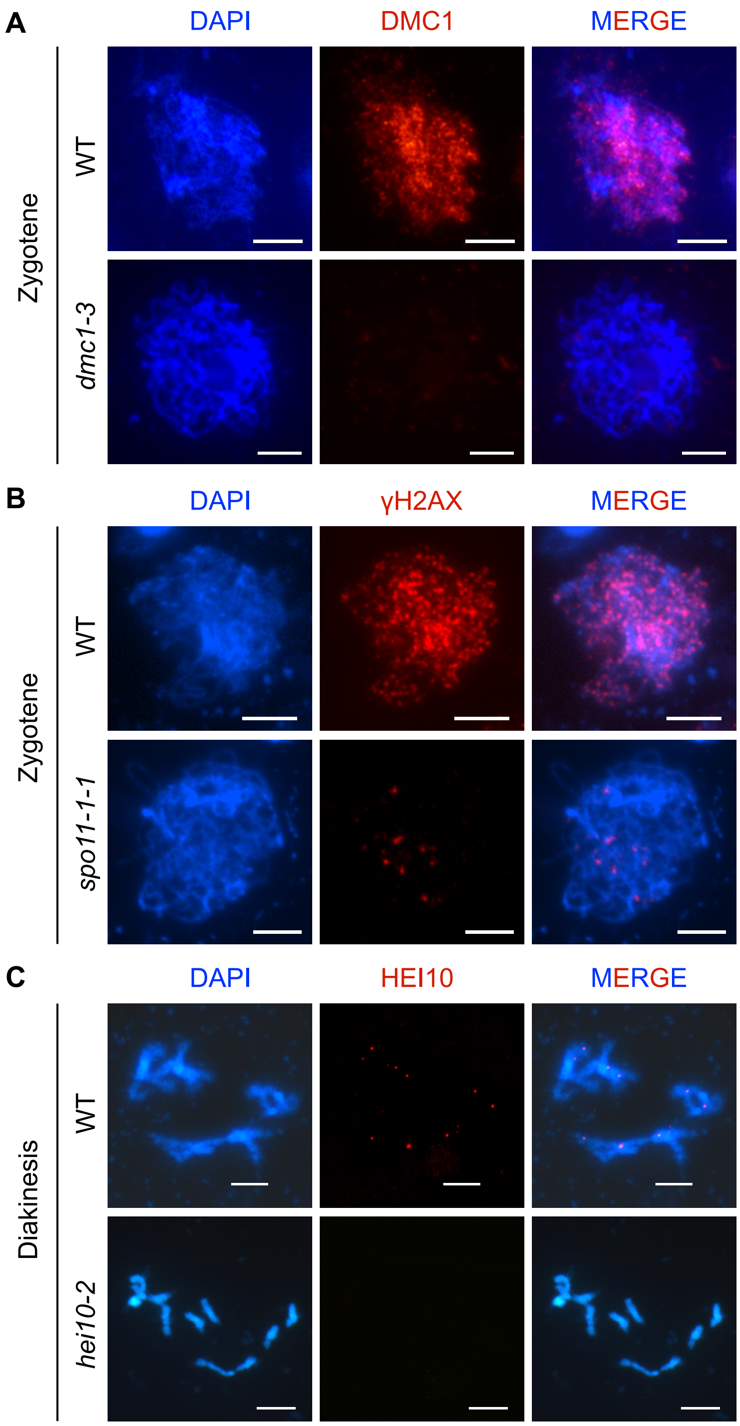

Figure. S7 Verification of antibodies used in this study through immunolocalization against wild type plants and corresponding mutants.

(A) Immunolocalization of DMC1 in zygotene meiocytes of wild type a nd *dmc1-3* mutant. **(B)** Immunolocalization of γH2AX in zygotene of wild type and *spo11-1-1* mutant. **(C)** Immunolocalization of HEI10 in diakinesis of wild type and *hei10-2* mutant.
