# Supplementary material for: ATM Promotes RAD51-Mediated Meiotic DSB Repair by Inter-Sister-Chromatid Recombination in Arabidopsis
Source: Front Plant Sci. 2020 Jun 25;11:839. doi: 10.3389/fpls.2020.00839 (PMC7329986; doi:10.3389/fpls.2020.00839)
Supplement: FIGURE S8 — Examples of metaphase I chromosome morphology for Figure 7A. Typical chromosome morphology of each type of metaphase I cells in different mutants exhibited in Figure 7A. [file Data_Sheet_8.PDF]

Figure S8

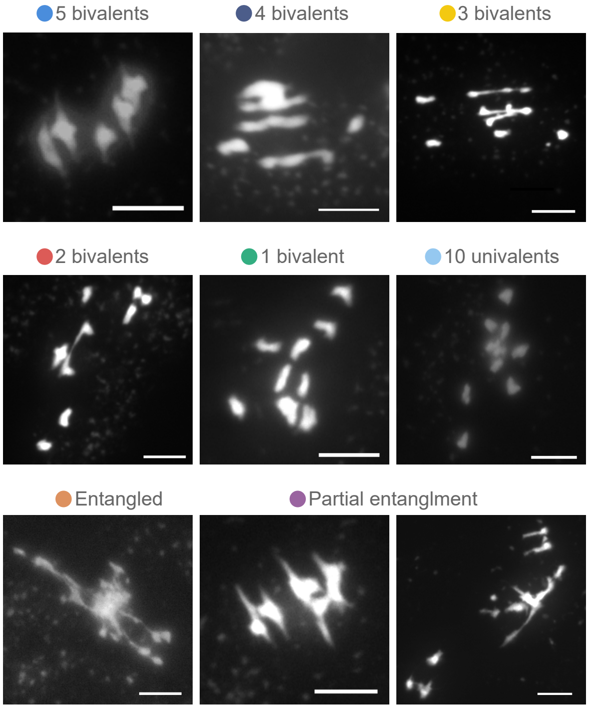

Figure. S8 Examples of metaphase I chromosome morphology for Figure 7A
Typical chromosome morphology of each type of metaphase I cells in different mutants exhibited in Figure 7A.
