# Supplementary material for: ATM Promotes RAD51-Mediated Meiotic DSB Repair by Inter-Sister-Chromatid Recombination in Arabidopsis
Source: Front Plant Sci. 2020 Jun 25;11:839. doi: 10.3389/fpls.2020.00839 (PMC7329986; doi:10.3389/fpls.2020.00839)
Supplement: TABLE S1 — List of primers used in this study. [file Table_1.DOCX]

**Table S1.** List of Primers Used in This Study

| **Name** | **Primer Sequences (5’-3’)** | **Purpose** |
| --- | --- | --- |
| oLF1971 | TCTCCGAGGAATCATCTGTTG | With oLF1972 for *atm-5* genomic band |
| oLF1972 | TTAGAAAACTGACCCGAAGGG |  |
| oLF249 | TTAGAAAACTGACCCGAAGGG | With oLF1972 for *atm-5* T-DNA insertion band |
| oLF1283 | TAGACGGATGATAACTATGACG | With oLF1284 for *dmc1* genomic band |
| oLF1284 | ACCCAGCGATTAAGCAACAA |  |
| oLF1285 | CCGGTATATCCCGTTTTCG | With oLF1284 for *dmc1* Ds insertion band |
| oLF1921 | GTTGCCGGATCAGCAGTAGT | With oLF1923 for *atm-4* genomic band |
| oLF1923 | GTGCCCTAGTCCAGTTTCCC |  |
| oLF1945 | AAGGCCGCATTGTGAAATCTT | With oLF1923 for *atm-4* insertion band |
| oLF2065 | GCAGCAAAAATTTCTTGGTTG | With oLF2066 for *atr-2* genomic band |
| oLF2066 | ACTTCAAGGGTTCCGATGTTC | With oLF249 for *atr-2* T-DNA insertion band |
| oLF1969 | ATCCATGTGGTTCAGTCTTGC | With oLF1970 for *atm-2* genomic band |
| oLF1970 | TTGGTATCCTGCAGAGGAAAG | With oLF249 for *atm-2* T-DNA insertion band |
| oLF1973 | CCAAACAAAATCGTTAGCCTG | With oLF1974 for *atm-3* genomic band |
| oLF1974 | CGAGGGTGTAGCCATATTCAC | With oLF249 for *atm-3* T-DNA insertion band |
| oLF1971 | TCTCCGAGGAATCATCTGTTG | With oLF1972 for *atm-5* genomic band |
| oLF1972 | TTAGAAAACTGACCCGAAGGG | With oLF249 for *atm-5* T-DNA insertion band |
| oLF2459 | ATGAACTTGGAAGGGTTACAAGA | With oLF2460 for *atm-1* genomic band |
| oLF2460 | TGGCAGCCGAGTATTTTTCAACTTT |  |
| oLF2461 | CACTCAGTCTTTCATCTACGGCA | With oLF2460 for *atm-1* T-DNA insertion band |
| oLF1928 | CCCTTTGGTTTATCAGAGCTGC | With oLF1929 for *spo11-1-1* genomic band |
| oLF1929 | CCAACGGCCCAACGTGTTTA |  |
| **Name** | **Primer Sequences (5’-3’)** | **Purpose** |
| oLF1930 | ACTGGGATTCGTCTTGGACA | With oLF1929 for *spo11-1-1* T-DNA insertion band |
| oLF1286 | ACTTCAGGCAGCAGGTATTGCTT | With oLF1287 for *rad51-3* genomic band |
| oLF1287 | TATACGCCCTCGCATAGGCA |  |
| oLF1288 | ACGATGGACTCCAGTCCGGCCGCTTCCTATTATATCTTCCCAAATTACCAATACA | With oLF1287 for *rad51-3* T-DNA insertion band |
| oLF1570 | TTCCGGTTTAAGCTGTATAGCTCTGACT | *ATM* genomic DNA segment 1 forward primer |
| oLF1571 | CAGGAAATTTAGTTTCTTCTGTTCGCTGAAC | *ATM* genomic DNA segment 1 reverse primer |
| oLF1572 | CGTGGCCCTTTTTGGTTAAACTTCTC | *ATM* genomic DNA segment 2 forward primer |
| oLF1573 | CTGGCAACCCATACGTGAAAAGTAACA | *ATM* genomic DNA segment 2 reverse primer |
| oLF1574 | AACCACATTACCGTTAAAATTCTGCCTCAAGA | *ATM* genomic DNA segment 3 forward primer |
| oLF1575 | CTCAATAAGGCCAGCTAAACTGACACC | *ATM* genomic DNA segment 3 reverse primer |
| oLF1576 | GAATTAATCATTGCCGCACTTGACAATTTATCA | *ATM* genomic DNA segment 4 forward primer |
| oLF1577 | CTTATTGGGACATCCTCACATAAAGCGA | *ATM* genomic DNA segment 4 reverse primer |
| oLF1578 | CCGAGAAATTTTCACTGGAGACGC | *ATM* genomic DNA segment 5 forward primer |
| oLF1579 | CTTGCGAAGTAATGAAGCAGACTGC | *ATM* genomic DNA segment 5 reverse primer |
| oLF1580 | TTGTCTTGGCTGAATAAGGATTGGAACTC | *ATM* genomic DNA segment 6 forward primer |
| oLF1581 | AGCATCTTGTCTGAGGTCATCATTGC | *ATM* genomic DNA segment 6 reverse primer |
| oLF1582 | AGTGATGAATGGTATAAATGCTCCAAAAGTAGTTG | *ATM* genomic DNA segment 7 forward primer |
| oLF1583 | ATAAAGTTCATAGCTGTATTGCGCTATCACTC | *ATM* genomic DNA segment 7 reverse primer |
| oLF1572 | CGTGGCCCTTTTTGGTTAAACTTCTC | *ATM* cDNA segment 1 forward primer |
| oLF1573 | CTGGCAACCCATACGTGAAAAGTAACA | *ATM* cDNA segment 1 reverse primer |
| oLF1574 | AACCACATTACCGTTAAAATTCTGCCTCAAGA | *ATM* cDNA segment 2 forward primer |
|  |  |  |
| **Name** | **Primer Sequences (5’-3’)** | **Purpose** |
| oLF1575 | CTCAATAAGGCCAGCTAAACTGACACC | *ATM* cDNA segment 2 reverse primer |
| oLF1576 | GAATTAATCATTGCCGCACTTGACAATTTATCA | *ATM* cDNA segment 3 forward primer |
| oLF1577 | CTTATTGGGACATCCTCACATAAAGCGA | *ATM* cDNA segment 3 reverse primer |
| oLF1578 | CCGAGAAATTTTCACTGGAGACGC | *ATM* cDNA segment 4 forward primer |
| oLF1579 | CTTGCGAAGTAATGAAGCAGACTGC | *ATM* cDNA segment 4 reverse primer |
| oLF1580 | TTGTCTTGGCTGAATAAGGATTGGAACTC | *ATM* cDNA segment 5 forward primer |
| oLF1581 | AGCATCTTGTCTGAGGTCATCATTGC | *ATM* cDNA segment 5 reverse primer |
| oLF1582 | AGTGATGAATGGTATAAATGCTCCAAAAGTAGTTG | *ATM* cDNA segment 6 forward primer |
| oLF1628 | TTACATCCAAGCTCCCCAGCCA | *ATM* cDNA segment 6 reverse primer |
| oLF2180 | AAGCCAAGACTCGCGAAGAT | *ATM* RT1 forward primer |
| oLF2181 | AAGGGCCACGTTTCAGCATT | *ATM* RT1 reverse primer |
| oLF2182 | GGATGTCTGTGAACAGATAGATGA | *ATM* RT2 forward primer |
| oLF2183 | CTACGTCAGGAACTTGGCAG | *ATM* RT2 reverse primer |
| oLF2184 | TTCCAGGAGTCTCCAAGCACT | *ATM* RT3 forward primer |
| oLF2185 | AGAGGCAGACACTCGGACAA | *ATM* RT3 reverse primer |
| oLF2186 | GCCGGATCAGCAGTAGTTTG | *ATM* RT4 forward primer |
| oLF2187 | CGTTTATTCTTGTTATTGCAGACAC | *ATM* RT4 reverse primer |
| oLF2188 | TTGCTTCAAGGTTGGGCAGT | *ATM* RT5 forward primer |
| oLF2189 | GTCACCGTTTGCCAAAGCC | *ATM* RT5 reverse primer |
| oLF2190 | GTCCCCCTTAAAGGCTTTGC | *ATM* RT6 forward primer |
| oLF2191 | CACCCTCGTAGCCATCAAGT | *ATM* RT6 reverse primer |
| oLF163 | GTTACCGACGGTTTTCATCC | Ds insertion right border specific primer |
| oLF164 | GGTCGGTACGGAATTCTCCC | Ds insertion left border specific primer |
| oLF3378 | GCGAGAAAATGATGGCTTCTCT | *DMC1* RT1 forward primer |
| oLF3379 | GTCAAGCACAGCTCCTGGAT | *DMC1* RT1 reverse primer |
| oLF3380 | CTTGCTTCTTGGCCTTGCTG | *DMC1* RT2 forward primer |
| oLF3381 | TCGCTCCTCTAATCCTTCGC | *DMC1* RT2 reverse primer |
