# Supplementary material for: ATM Promotes RAD51-Mediated Meiotic DSB Repair by Inter-Sister-Chromatid Recombination in Arabidopsis
Source: Front Plant Sci. 2020 Jun 25;11:839. doi: 10.3389/fpls.2020.00839 (PMC7329986; doi:10.3389/fpls.2020.00839)
Supplement: TABLE S2 — List of SSLP markers primers used in this study. [file Table_2.DOCX]

**Table S2.** **List of SSLP Marker Primers Used in This Study**

| **Marker** | **Chr.** | **Position (Mbp)** | **Col-0** | **L*er*** | **Forward Primer Reverse Primer** |
| --- | --- | --- | --- | --- | --- |
| NF7G19 | 1 | 2.85 | 197 | >197 | TCGTTGAAAACGATTAGATTGG |
|  |  |  |  |  | TTCAAAAATCGTGAGATGAAATG |
| F20D23 | 1 | 5.889 | 233 | +21 | TTATGCCAACTCATGTGGAAAG |
|  |  |  |  |  | TGTCAAAGCGTCTGGTTCTG |
| F12K8 | 1 | 7.954 | 168 | +37 | ACCAACACCACAACAAACGAC |
|  |  |  |  |  | CTTTTTCTGTTCTTCCGCTATTC |
| F13K9-30476 | 1 | 9.744 | 188 | -27 | GGTAGATGCCAATGGAGGAA |
|  |  |  |  |  | TTCACATGTTTCAGGCGAAC |
| F6N18-69636 | 1 | 11.859 | 164 | -21 | TGCTCGGAAAGTAAAAGTTGG |
|  |  |  |  |  | TGGTTCGGTTGGATTTGTTT |
| **Centromere** | | | | | |
| T10P12-20470 | 1 | 16.373 | 223 | -23 | TATTGTGTTCCCACCAAACG |
|  |  |  |  |  | TGTTGGGTTGCTATGCATGT |
| ciw1 | 1 | 18.367 | 159 | -24 | ACATTTTCTCAATCCTTACTC |
|  |  |  |  |  | GAGAGCTTCTTTATTTGTGAT |
| NF11P17 | 1 | 22.602 | 209 | <209 | TTTCAGTTTGATGATTTATTCGC |
|  |  |  |  |  | CGCAATCGATTTTATTTAAATCC |
| F20P5 | 1 | 26.464 | 218 | -38 | GATACGTTCAAAATTAGGGACTTC |
|  |  |  |  |  | TGTATTTTGCTAATTGAGGTTATGG |
| F3F9-74814 | 1 | 29.493 | 187 | -35 | CGTAACATATCTTCATTCGCCTTT |
|  |  |  |  |  | TTTCTGGTCAACTGAAATCCA |
| T17M13 | 2 | 0.830 | 336 | -43 | CTGGAGATCATCCAACAAAG |
|  |  |  |  |  | TGCAATGGAATGGGCTGGTC |
| F15L11-12250 | 2 | 1.830 | 253 | -43 | AGAGGCTGATCGGTCTGAAA |
|  |  |  |  |  | GCGGGTGTTACGATAGAGGA |
| **Centromere** | | | | | |
| ciw3 | 2 | 6.409 | 230 | -30 | GAAACTCAATGAAATCCACTT |
|  |  |  |  |  | TGAACTTGTTGTGAGCTTTGA |
| F26B6 | 2 | 10.000 | 223 | +51 | CTCTATCTGCCCACGAACAAG |
|  |  |  |  |  | GCCATTGCAAAAGAACATCAG |
| T9D9-46843 | 2 | 12.937 | 272 | -45 | GAATTTCTCAATTTTCAGGACTAACA |
|  |  |  |  |  | CGAGATTGAATGGTGATCCA |
| T1J8-46260 | 2 | 15.492 | 226 | -26 | CGGGCTGTCCATGAACTATT |
|  |  |  |  |  | AAACCAAATCGAACCAACCA |
| F4I18-34837 | 2 | 18.899 | 290 | -43 | GACCCGAGGGTTATATGCAA |
|  |  |  |  |  | CCGCAGCCATGCTTATTTTA |
| F13E7-68449 | 3 | 0.617 | 239 | -30 | TCGACTCCAGTCCAAATGTTC |
|  |  |  |  |  | TACGCGTGTTTCCCCTTTAC |
| nga162 | 3 | 4.608 | 146 | -18 | GCAATTTGCATCTGAGGAAT |
|  |  |  |  |  | GCTCCTGAGTTTCGGACAGA |
| **Marker** | **Chr.** | **Position (Mbp)** | **Col-0** | **L*er*** | **Forward Primer Reverse Primer** |
| MSA6-50838 | 3 | 7.405 | 227 | -41 | CCCTGGCAAGACATAACCAA |
|  |  |  |  |  | CTTCTTGTTTTGCCTCTGTGG |
| ciw11a | 3 | 9.775 | 192 | +50 | GTTTTTTCTAATCCCCGAGTTGAG |
|  |  |  |  |  | GAAGAAATTCCTAAAGCATTC |
| T13B17-321 | 3 | 11.329 | 202 | -48 | TTTAACAGATTTTAGGAAAACAAATCA |
|  |  |  |  |  | TTGGTAAACAAACCATCACCTTT |
| **Centromere** | | | | | |
| T4P3-6097 | 3 | 14.137 | 193 | -25 | CACATTCTCGAGGTGCACTG |
|  |  |  |  |  | GTTACCGCACAAGAGGTCGT |
| F13I12-58714v2 | 3 | 17.350 | 232 | -26 | TGGACCCAAGTCTTTGGATT |
|  |  |  |  |  | CCCTCGTTTCTCTTTCTCGTT |
| MSAT3.10 | 3 | 17.260 | 293 | -33 | CTCCATTGGGCAGAGAGAAC |
|  |  |  |  |  | TGGCATTGTCCCTATGGG |
| F13I13 | 3 | 17.341 | 232 | -26 | TGGACCCAAGTCTTTGGATT |
|  |  |  |  |  | CCCTCGTTTCTCTTTCTCGTT |
| T23J7-19 | 3 | 17.593 | 148 | 19 | ATAACTTCCACAATGCTGGTTCTTC |
|  |  |  |  |  | ACTACTATCAGTGTGACACATCCAA |
| 7558 | 3 | 17.730 | 101 | -6 | CGTAGTTTGCATGATATGAA |
|  |  |  |  |  | GCTCATTTTGTTACAAATAG |
| 7612 | 3 | 17.856 | 169 | -32 | GCACACATTTGTTATGTTAT |
|  |  |  |  |  | TAGCCGAACCGCACCTTT |
| PDE312-14 | 3 | 17.965 | 139 | 14 | TCTTACACTGGCGCCGATCTT |
|  |  |  |  |  | GTGCTTGCCGACTGCAAAATA |
| F2K15-49 | 3 | 18.295 | 364 | 49 | ATTTATCGGAGGGTGAGCTT |
|  |  |  |  |  | AACACAACTCATGTCTGTTTG |
| ciw4 | 3 | 18.901 | 189 | +26 | GTTCATTAAACTTGCGTGTGT |
|  |  |  |  |  | TACGGTCAGATTGAGTGATTC |
| 8388 | 3 | 19.679 | 141 | -30 | TTGGAGTTCTACGAAGAAGT |
|  |  |  |  |  | TTAGAGGAAGGAAGCGAATA |
| MSAT3.29 | 3 | 20.487 | 238 | -18 | CGGATGAGATCCAA |
|  |  |  |  |  | GACAGAGGTTTACTAATGT |
| F27K19 | 3 | 20.820 | 237 | +44 | TGCTTTTGAAGAGATGGTTATTAGG |
|  |  |  |  |  | CCCCATTTCACTTATCATTGG |
| T20O10-24167 | 3 | 23.292 | 215 | -25 | GGGGAAACAGATAAGGAAGCA |
|  |  |  |  |  | CGCTGTAGCAAACGTGGTAA |
| ciw5 | 4 | 0.738 | 164 | -20 | GGTTAAAAATTAGGGTTACGA |
|  |  |  |  |  | AGATTTACGTGGAAGCAAT |
| C17L7-38326 | 4 | 2.683 | 217 | -25 | TGAAGCGGTTTCAAGATTGTT |
|  |  |  |  |  | CGCAAACTATTTGCCATAGTCA |
| **Centromere** | | | | | |
| T12G13-90376 | 4 | 5.253 | 168 | -26 | CGATTTCGGTTCGCATCTA |
|  |  |  |  |  | ACCCGAACCCAAAACTAAAAA |
| **Marker** | **Chr.** | **Position (Mbp)** | **Col-0** | **L*er*** | **Forward Primer Reverse Primer** |
| T26M18 | 4 | 7.157 | 330 | -59 | ACATTAGCGGAGGCCACTTC |
|  |  |  |  |  | GGGCAAAAGCTTCCAGTAC |
| F28A21-47297 | 4 | 10.288 | 207 | -29 | GCAATAAAAGAAAGGGGGAAA |
|  |  |  |  |  | TGACAATCACGGATACAAAAGC |
| F26K10 | 4 | 13.991 | 180 | +25 | AGAGAGCACGATGCCTGATAG |
|  |  |  |  |  | AATGCTTCAGCGATTGAGAAC |
| F23E13 | 4 | 17.148 | 264 | -39 | TGACCGTTGAAAGTGTTGTTG |
|  |  |  |  |  | GCCCGAGAAGCCTGATAG |
| T5J17-51431 | 4 | 18.513 | 193 | -19 | GCACACAGAGATATCGAAAAATCA |
|  |  |  |  |  | GCGTTTGATTTCAAATTCGTT |
| MOJB | 5 | 2.190 | 178 | -20 | GAAGATGAAAGATTTTAGGAGGAC |
|  |  |  |  |  | GTTTGTAGGAGAAGGGGACAAG |
| F5E19-2891 | 5 | 5.469 | 192 | -17 | GACTAAACTGGCATTAAGTTCTGAG |
|  |  |  |  |  | TCGTAGTTCGTACCTGTGCCTA |
| MRO11-71713 | 5 | 8.013 | 100 | +35 | CGTTATGTAATAGTCATCACGTTTTTG |
|  |  |  |  |  | TTCACAATTAGAACGCTGAATCAT |
| nga76 | 5 | 10.419 | 220 | +80 | AGGCATGGGAGACATTTACG |
|  |  |  |  |  | GGAGAAAATGTCACTCTCCACC |
| **Centromere** | | | | | |
| phyC.3 | 5 | 14.025 | 211 | +15 | AAACTCGAGAGTTTTGTCTAG |
|  |  |  |  |  | CTCAGAGAATTCCCAGAAAAATC |
| MFO20-693 | 5 | 17.059 | 228 | -42 | AACTATGTTTGGCATTTAAAGGTT |
|  |  |  |  |  | AGCAAATGGAACCGAAACAA |
| K9P8 | 5 | 20.312 | 284 | -33 | TTATGGGTTTCTCAGAGTTTCTCAC |
|  |  |  |  |  | TTGTATGCGTTTGCTTTTTCC |
| MBG8-3386 | 5 | 22.262 | 197 | -39 | TGAACTGGATCAGCTTTACTTGA |
|  |  |  |  |  | AGGTGCACGAGATGGTTCAT |
| MQB2 | 5 | 25.208 | 252 | -21 | CTTTGATAGTAACCTTTTTCAAACCA |
|  |  |  |  |  | TGCCATTTATTTGGTCAACAC |
