# Supplementary material for: ATM Promotes RAD51-Mediated Meiotic DSB Repair by Inter-Sister-Chromatid Recombination in Arabidopsis
Source: Front Plant Sci. 2020 Jun 25;11:839. doi: 10.3389/fpls.2020.00839 (PMC7329986; doi:10.3389/fpls.2020.00839)
Supplement: TABLE S3 — Numbers of counted γH2AX signal foci in each image. [file Table_3.DOCX]

**Table S3. Numbers of counted γH2AX signal foci in each image.**

| **Allele** | **Stage** | **Foci** | **Allele** | **Stage** | **Foci** | **Allele** | **Stage** | **Foci** | **Allele** | **Stage** | **Foci** | **Allele** | **Stage** | **Foci** |
| --- | --- | --- | --- | --- | --- | --- | --- | --- | --- | --- | --- | --- | --- | --- |
| WT | Zygotene | 220 | *atm-2* | Zygotene | 154 | *atm-5* | Zygotene | 162 | *atm-5 atr-2* | Zygotene | 37 | *atr-2* | Zygotene | 213 |
| WT | Zygotene | 220 | *atm-2* | Zygotene | 172 | *atm-5* | Zygotene | 161 | *atm-5 atr-2* | Zygotene | 30 | *atr-2* | Zygotene | 177 |
| WT | Zygotene | 219 | *atm-2* | Zygotene | 136 | *atm-5* | Zygotene | 160 | *atm-5 atr-2* | Zygotene | 30 | *atr-2* | Zygotene | 175 |
| WT | Zygotene | 217 | *atm-2* | Zygotene | 138 | *atm-5* | Zygotene | 160 | *atm-5 atr-2* | Zygotene | 29 | *atr-2* | Zygotene | 192 |
| WT | Zygotene | 215 | *atm-2* | Zygotene | 160 | *atm-5* | Zygotene | 160 | *atm-5 atr-2* | Zygotene | 28 | *atr-2* | Zygotene | 213 |
| WT | Zygotene | 215 | *atm-2* | Zygotene | 149 | *atm-5* | Zygotene | 159 | *atm-5 atr-2* | Zygotene | 26 | *atr-2* | Zygotene | 245 |
| WT | Zygotene | 215 | *atm-2* | Zygotene | 131 | *atm-5* | Zygotene | 159 | *atm-5 atr-2* | Zygotene | 25 | *atr-2* | Zygotene | 235 |
| WT | Zygotene | 215 | *atm-2* | Zygotene | 112 | *atm-5* | Zygotene | 159 | *atm-5 atr-2* | Zygotene | 25 | *atr-2* | Zygotene | 178 |
| WT | Zygotene | 215 | *atm-2* | Zygotene | 125 | *atm-5* | Zygotene | 158 | *atm-5 atr-2* | Zygotene | 24 | *atr-2* | Zygotene | 206 |
| WT | Zygotene | 214 | *atm-2* | Zygotene | 148 | *atm-5* | Zygotene | 157 | *atm-5 atr-2* | Zygotene | 24 | *atr-2* | Zygotene | 178 |
| WT | Zygotene | 208 | *atm-2* | Zygotene | 156 | *atm-5* | Zygotene | 157 | *atm-5 atr-2* | Zygotene | 23 | *atr-2* | Zygotene | 202 |
| WT | Zygotene | 206 | *atm-2* | Zygotene | 135 | *atm-5* | Zygotene | 156 | *atm-5 atr-2* | Zygotene | 23 | *atr-2* | Zygotene | 228 |
| WT | Zygotene | 206 | *atm-2* | Zygotene | 155 | *atm-5* | Zygotene | 154 | *atm-5 atr-2* | Zygotene | 23 | *atr-2* | Zygotene | 224 |
| WT | Zygotene | 206 | *atm-2* | Zygotene | 156 | *atm-5* | Zygotene | 153 | *atm-5 atr-2* | Zygotene | 22 | *atr-2* | Zygotene | 187 |
| WT | Zygotene | 206 | *atm-2* | Zygotene | 143 | *atm-5* | Zygotene | 153 | *atm-5 atr-2* | Zygotene | 22 | *atr-2* | Zygotene | 173 |
| WT | Zygotene | 205 | *atm-2* | Zygotene | 118 | *atm-5* | Zygotene | 151 | *atm-5 atr-2* | Zygotene | 21 | *atr-2* | Zygotene | 214 |
| WT | Zygotene | 202 | *atm-2* | Zygotene | 137 | *atm-5* | Zygotene | 151 | *atm-5 atr-2* | Zygotene | 21 | *atr-2* | Zygotene | 222 |
| WT | Zygotene | 200 | *atm-2* | Zygotene | 143 | *atm-5* | Zygotene | 150 | *atm-5 atr-2* | Zygotene | 20 | *atr-2* | Zygotene | 247 |
| WT | Zygotene | 200 | *atm-2* | Zygotene | 132 | *atm-5* | Zygotene | 150 | *atm-5 atr-2* | Zygotene | 19 | *atr-2* | Zygotene | 197 |
| WT | Zygotene | 199 | *atm-2* | Zygotene | 167 | *atm-5* | Zygotene | 150 | *atm-5 atr-2* | Zygotene | 18 | *atr-2* | Zygotene | 215 |
| WT | Zygotene | 197 | *atm-2* | Zygotene | 181 | *atm-5* | Zygotene | 150 | *atm-5 atr-2* | Zygotene | 18 | *atr-2* | Zygotene | 202 |
| WT | Zygotene | 194 | *atm-2* | Zygotene | 155 | *atm-5* | Zygotene | 149 | *atm-5 atr-2* | Zygotene | 16 | *atr-2* | Zygotene | 249 |
| WT | Zygotene | 193 | *atm-2* | Zygotene | 144 | *atm-5* | Zygotene | 148 | *atm-5 atr-2* | Zygotene | 16 | *atr-2* | Zygotene | 175 |
| WT | Zygotene | 193 | *atm-2* | Zygotene | 172 | *atm-5* | Zygotene | 148 | *atm-5 atr-2* | Zygotene | 16 | *atr-2* | Zygotene | 208 |
| WT | Zygotene | 193 | *atm-2* | Zygotene | 118 | *atm-5* | Zygotene | 144 | *atm-5 atr-2* | Zygotene | 13 | *atr-2* | Zygotene | 274 |
| WT | Zygotene | 192 | *atm-2* | Zygotene | 125 | *atm-5* | Zygotene | 144 | *atm-5 atr-2* | Zygotene | 12 | *atr-2* | Zygotene | 217 |
| WT | Zygotene | 192 | *atm-2* | Zygotene | 170 | *atm-5* | Zygotene | 143 | *atm-5 atr-2* | Zygotene | 12 | *atr-2* | Zygotene | 221 |
| WT | Zygotene | 191 | *atm-2* | Zygotene | 177 | *atm-5* | Zygotene | 142 | *atm-5 atr-2* | Zygotene | 11 | *atr-2* | Zygotene | 200 |
| WT | Zygotene | 191 | *atm-2* | Zygotene | 148 | *atm-5* | Zygotene | 141 | *atm-5 atr-2* | Zygotene | 8 | *atr-2* | Zygotene | 200 |
| WT | Zygotene | 183 | *atm-2* | Zygotene | 144 | *atm-5* | Zygotene | 140 |  |  |  | *atr-2* | Zygotene | 222 |
|  |  |  | *atm-2* | Zygotene | 172 |  |  |  |  |  |  | *atr-2* | Zygotene | 208 |
|  |  |  | *atm-2* | Zygotene | 126 |  |  |  |  |  |  | *atr-2* | Zygotene | 170 |
|  |  |  |  |  |  |  |  |  |  |  |  | *atr-2* | Zygotene | 213 |
| **Allele** | **Stage** | **Foci** | **Allele** | **Stage** | **Foci** | **Allele** | **Stage** | **Foci** | **Allele** | **Stage** | **Foci** | **Allele** | **Stage** | **Foci** |
| WT | Pachytene | 98 | *atm-2* | Pachytene | 54 | *atm-5* | Pachytene | 57 | *atm-5 atr-2* | Pachytene | 30 | *atr-2* | Pachytene | 90 |
| WT | Pachytene | 97 | *atm-2* | Pachytene | 40 | *atm-5* | Pachytene | 57 | *atm-5 atr-2* | Pachytene | 28 | *atr-2* | Pachytene | 95 |
| WT | Pachytene | 97 | *atm-2* | Pachytene | 66 | *atm-5* | Pachytene | 57 | *atm-5 atr-2* | Pachytene | 27 | *atr-2* | Pachytene | 85 |
| WT | Pachytene | 96 | *atm-2* | Pachytene | 71 | *atm-5* | Pachytene | 57 | *atm-5 atr-2* | Pachytene | 26 | *atr-2* | Pachytene | 104 |
| WT | Pachytene | 91 | *atm-2* | Pachytene | 55 | *atm-5* | Pachytene | 57 | *atm-5 atr-2* | Pachytene | 26 | *atr-2* | Pachytene | 79 |
| WT | Pachytene | 91 | *atm-2* | Pachytene | 49 | *atm-5* | Pachytene | 56 | *atm-5 atr-2* | Pachytene | 25 | *atr-2* | Pachytene | 63 |
| WT | Pachytene | 89 | *atm-2* | Pachytene | 66 | *atm-5* | Pachytene | 56 | *atm-5 atr-2* | Pachytene | 25 | *atr-2* | Pachytene | 67 |
| WT | Pachytene | 88 | *atm-2* | Pachytene | 40 | *atm-5* | Pachytene | 55 | *atm-5 atr-2* | Pachytene | 24 | *atr-2* | Pachytene | 90 |
| WT | Pachytene | 86 | *atm-2* | Pachytene | 49 | *atm-5* | Pachytene | 55 | *atm-5 atr-2* | Pachytene | 24 | *atr-2* | Pachytene | 70 |
| WT | Pachytene | 86 | *atm-2* | Pachytene | 51 | *atm-5* | Pachytene | 54 | *atm-5 atr-2* | Pachytene | 24 | *atr-2* | Pachytene | 57 |
| WT | Pachytene | 84 | *atm-2* | Pachytene | 41 | *atm-5* | Pachytene | 54 | *atm-5 atr-2* | Pachytene | 23 | *atr-2* | Pachytene | 77 |
| WT | Pachytene | 83 | *atm-2* | Pachytene | 47 | *atm-5* | Pachytene | 53 | *atm-5 atr-2* | Pachytene | 23 | *atr-2* | Pachytene | 60 |
| WT | Pachytene | 82 | *atm-2* | Pachytene | 54 | *atm-5* | Pachytene | 53 | *atm-5 atr-2* | Pachytene | 21 | *atr-2* | Pachytene | 63 |
| WT | Pachytene | 80 | *atm-2* | Pachytene | 39 | *atm-5* | Pachytene | 53 | *atm-5 atr-2* | Pachytene | 21 | *atr-2* | Pachytene | 88 |
| WT | Pachytene | 80 | *atm-2* | Pachytene | 41 | *atm-5* | Pachytene | 53 | *atm-5 atr-2* | Pachytene | 21 | *atr-2* | Pachytene | 71 |
| WT | Pachytene | 79 | *atm-2* | Pachytene | 25 | *atm-5* | Pachytene | 52 | *atm-5 atr-2* | Pachytene | 19 | *atr-2* | Pachytene | 57 |
| WT | Pachytene | 78 | *atm-2* | Pachytene | 52 | *atm-5* | Pachytene | 52 | *atm-5 atr-2* | Pachytene | 18 | *atr-2* | Pachytene | 50 |
| WT | Pachytene | 78 | *atm-2* | Pachytene | 51 | *atm-5* | Pachytene | 51 | *atm-5 atr-2* | Pachytene | 18 | *atr-2* | Pachytene | 61 |
| WT | Pachytene | 77 | *atm-2* | Pachytene | 42 | *atm-5* | Pachytene | 50 | *atm-5 atr-2* | Pachytene | 17 | *atr-2* | Pachytene | 65 |
| WT | Pachytene | 77 | *atm-2* | Pachytene | 46 | *atm-5* | Pachytene | 50 | *atm-5 atr-2* | Pachytene | 17 | *atr-2* | Pachytene | 68 |
| WT | Pachytene | 76 | *atm-2* | Pachytene | 48 | *atm-5* | Pachytene | 49 | *atm-5 atr-2* | Pachytene | 17 | *atr-2* | Pachytene | 72 |
| WT | Pachytene | 74 | *atm-2* | Pachytene | 35 | *atm-5* | Pachytene | 49 | *atm-5 atr-2* | Pachytene | 17 | *atr-2* | Pachytene | 91 |
| WT | Pachytene | 72 | *atm-2* | Pachytene | 42 | *atm-5* | Pachytene | 48 | *atm-5 atr-2* | Pachytene | 16 | *atr-2* | Pachytene | 85 |
| WT | Pachytene | 71 | *atm-2* | Pachytene | 57 | *atm-5* | Pachytene | 48 | *atm-5 atr-2* | Pachytene | 16 | *atr-2* | Pachytene | 65 |
| WT | Pachytene | 70 | *atm-2* | Pachytene | 52 | *atm-5* | Pachytene | 48 | *atm-5 atr-2* | Pachytene | 16 | *atr-2* | Pachytene | 51 |
| WT | Pachytene | 67 | *atm-2* | Pachytene | 53 | *atm-5* | Pachytene | 47 | *atm-5 atr-2* | Pachytene | 15 | *atr-2* | Pachytene | 107 |
| WT | Pachytene | 67 | *atm-2* | Pachytene | 46 | *atm-5* | Pachytene | 43 | *atm-5 atr-2* | Pachytene | 15 | *atr-2* | Pachytene | 50 |
| WT | Pachytene | 66 | *atm-2* | Pachytene | 39 | *atm-5* | Pachytene | 40 | *atm-5 atr-2* | Pachytene | 15 | *atr-2* | Pachytene | 51 |
| WT | Pachytene | 64 | *atm-2* | Pachytene | 34 | *atm-5* | Pachytene | 39 | *atm-5 atr-2* | Pachytene | 13 | *atr-2* | Pachytene | 63 |
| WT | Pachytene | 62 | *atm-2* | Pachytene | 35 | *atm-5* | Pachytene | 39 | *atm-5 atr-2* | Pachytene | 13 | *atr-2* | Pachytene | 65 |
|  |  |  | *atm-2* | Pachytene | 41 |  |  |  | *atm-5 atr-2* | Pachytene | 12 | *atr-2* | Pachytene | 74 |
|  |  |  |  |  |  |  |  |  | *atm-5 atr-2* | Pachytene | 12 | *atr-2* | Pachytene | 84 |
|  |  |  |  |  |  |  |  |  | *atm-5 atr-2* | Pachytene | 10 |  |  |  |
|  |  |  |  |  |  |  |  |  | *atm-5 atr-2* | Pachytene | 10 |  |  |  |
|  |  |  |  |  |  |  |  |  | *atm-5 atr-2* | Pachytene | 10 |  |  |  |
|  |  |  |  |  |  |  |  |  | *atm-5 atr-2* | Pachytene | 7 |  |  |  |
