# Supplementary material for: ATM Promotes RAD51-Mediated Meiotic DSB Repair by Inter-Sister-Chromatid Recombination in Arabidopsis
Source: Front Plant Sci. 2020 Jun 25;11:839. doi: 10.3389/fpls.2020.00839 (PMC7329986; doi:10.3389/fpls.2020.00839)
Supplement: TABLE S4 — Numbers of counted DMC1 immunolocalization signal foci in each image. [file Table_4.DOCX]

**Table S4. Numbers of counted DMC1 immunolocalization signal foci in each image.**

| **Allele** | **Stage** | **Foci** | ***Allele*** | **Stage** | **Foci** | **Allele** | **Stage** | **Foci** |
| --- | --- | --- | --- | --- | --- | --- | --- | --- |
| WT | Zygotene | 283 | *atm-5* | Zygotene | 304 | *atm-2* | Zygotene | 241 |
| WT | Zygotene | 280 | *atm-5* | Zygotene | 303 | *atm-2* | Zygotene | 200 |
| WT | Zygotene | 275 | *atm-5* | Zygotene | 300 | *atm-2* | Zygotene | 275 |
| WT | Zygotene | 255 | *atm-5* | Zygotene | 297 | *atm-2* | Zygotene | 188 |
| WT | Zygotene | 242 | *atm-5* | Zygotene | 293 | *atm-2* | Zygotene | 265 |
| WT | Zygotene | 241 | *atm-5* | Zygotene | 273 | *atm-2* | Zygotene | 184 |
| WT | Zygotene | 239 | *atm-5* | Zygotene | 270 | *atm-2* | Zygotene | 246 |
| WT | Zygotene | 239 | *atm-5* | Zygotene | 270 | *atm-2* | Zygotene | 233 |
| WT | Zygotene | 235 | *atm-5* | Zygotene | 264 | *atm-2* | Zygotene | 218 |
| WT | Zygotene | 223 | *atm-5* | Zygotene | 256 | *atm-2* | Zygotene | 218 |
| WT | Zygotene | 218 | *atm-5* | Zygotene | 246 | *atm-2* | Zygotene | 228 |
| WT | Zygotene | 217 | *atm-5* | Zygotene | 244 | *atm-2* | Zygotene | 223 |
| WT | Zygotene | 217 | *atm-5* | Zygotene | 243 | *atm-2* | Zygotene | 165 |
| WT | Zygotene | 214 | *atm-5* | Zygotene | 243 | *atm-2* | Zygotene | 263 |
| WT | Zygotene | 201 | *atm-5* | Zygotene | 238 | *atm-2* | Zygotene | 235 |
| WT | Zygotene | 200 | *atm-5* | Zygotene | 230 | *atm-2* | Zygotene | 249 |
| WT | Zygotene | 195 | *atm-5* | Zygotene | 224 | *atm-2* | Zygotene | 214 |
| WT | Zygotene | 188 | *atm-5* | Zygotene | 221 | *atm-2* | Zygotene | 216 |
| WT | Zygotene | 186 | *atm-5* | Zygotene | 218 | *atm-2* | Zygotene | 243 |
| WT | Zygotene | 180 | *atm-5* | Zygotene | 217 | *atm-2* | Zygotene | 216 |
| WT | Zygotene | 177 | *atm-5* | Zygotene | 214 | *atm-2* | Zygotene | 215 |
| WT | Zygotene | 177 | *atm-5* | Zygotene | 210 | *atm-2* | Zygotene | 200 |
| WT | Zygotene | 175 | *atm-5* | Zygotene | 201 | *atm-2* | Zygotene | 250 |
| WT | Zygotene | 173 | *atm-5* | Zygotene | 196 | *atm-2* | Zygotene | 255 |
| WT | Zygotene | 172 | *atm-5* | Zygotene | 191 | *atm-2* | Zygotene | 227 |
| WT | Zygotene | 167 | *atm-5* | Zygotene | 185 | *atm-2* | Zygotene | 237 |
| WT | Zygotene | 167 | *atm-5* | Zygotene | 172 | *atm-2* | Zygotene | 211 |
| WT | Zygotene | 149 | *atm-5* | Zygotene | 166 | *atm-2* | Zygotene | 162 |
| WT | Zygotene | 145 | *atm-5* | Zygotene | 160 | *atm-2* | Zygotene | 199 |
| WT | Zygotene | 144 |  |  |  | *atm-2* | Zygotene | 195 |
| WT | Zygotene | 144 |  |  |  | *atm-2* | Zygotene | 260 |
| WT | Zygotene | 142 |  |  |  | *atm-2* | Zygotene | 216 |
| WT | Zygotene | 140 |  |  |  |  |  |  |
| WT | Zygotene | 137 |  |  |  |  |  |  |
| WT | Zygotene | 135 |  |  |  |  |  |  |
| **Allele** | **Stage** | **Foci** | **Allele** | **Stage** | **Foci** | **Allele** | **Stage** | **Foci** |
| WT | Pachytene | 56 | *atm-5* | Pachytene | 60 | *atm-2* | Pachytene | 52 |
| WT | Pachytene | 53 | *atm-5* | Pachytene | 54 | *atm-2* | Pachytene | 76 |
| WT | Pachytene | 54 | *atm-5* | Pachytene | 57 | *atm-2* | Pachytene | 75 |
| WT | Pachytene | 44 | *atm-5* | Pachytene | 55 | *atm-2* | Pachytene | 45 |
| WT | Pachytene | 45 | *atm-5* | Pachytene | 55 | *atm-2* | Pachytene | 43 |
| WT | Pachytene | 40 | *atm-5* | Pachytene | 63 | *atm-2* | Pachytene | 68 |
| WT | Pachytene | 46 | *atm-5* | Pachytene | 58 | *atm-2* | Pachytene | 52 |
| WT | Pachytene | 40 | *atm-5* | Pachytene | 45 | *atm-2* | Pachytene | 47 |
| WT | Pachytene | 55 | *atm-5* | Pachytene | 46 | *atm-2* | Pachytene | 60 |
| WT | Pachytene | 46 | *atm-5* | Pachytene | 58 | *atm-2* | Pachytene | 56 |
| WT | Pachytene | 53 | *atm-5* | Pachytene | 46 | *atm-2* | Pachytene | 45 |
| WT | Pachytene | 45 | *atm-5* | Pachytene | 69 | *atm-2* | Pachytene | 65 |
| WT | Pachytene | 63 | *atm-5* | Pachytene | 70 | *atm-2* | Pachytene | 51 |
| WT | Pachytene | 68 | *atm-5* | Pachytene | 42 | *atm-2* | Pachytene | 55 |
| WT | Pachytene | 49 | *atm-5* | Pachytene | 75 | *atm-2* | Pachytene | 65 |
| WT | Pachytene | 62 | *atm-5* | Pachytene | 43 | *atm-2* | Pachytene | 50 |
| WT | Pachytene | 42 | *atm-5* | Pachytene | 54 | *atm-2* | Pachytene | 59 |
| WT | Pachytene | 65 | *atm-5* | Pachytene | 49 | *atm-2* | Pachytene | 53 |
| WT | Pachytene | 40 | *atm-5* | Pachytene | 34 | *atm-2* | Pachytene | 53 |
| WT | Pachytene | 49 | *atm-5* | Pachytene | 48 | *atm-2* | Pachytene | 52 |
| WT | Pachytene | 46 | *atm-5* | Pachytene | 55 | *atm-2* | Pachytene | 48 |
| WT | Pachytene | 46 | *atm-5* | Pachytene | 48 | *atm-2* | Pachytene | 49 |
| WT | Pachytene | 35 | *atm-5* | Pachytene | 65 | *atm-2* | Pachytene | 57 |
| WT | Pachytene | 53 | *atm-5* | Pachytene | 48 | *atm-2* | Pachytene | 60 |
|  |  |  | *atm-5* | Pachytene | 63 | *atm-2* | Pachytene | 58 |
|  |  |  |  |  |  | *atm-2* | Pachytene | 60 |
|  |  |  |  |  |  | *atm-2* | Pachytene | 44 |
