# Supplementary material for: ATM Promotes RAD51-Mediated Meiotic DSB Repair by Inter-Sister-Chromatid Recombination in Arabidopsis
Source: Front Plant Sci. 2020 Jun 25;11:839. doi: 10.3389/fpls.2020.00839 (PMC7329986; doi:10.3389/fpls.2020.00839)
Supplement: TABLE S5 — Numbers of counted RAD51 immunolocalization signal foci in each image. [file Table_5.DOCX]

**Table S5. Numbers of counted RAD51 immunolocalization signal foci in each image.**

| **Allele** | **Stage** | **Foci** | **Allele** | **Stage** | **Foci** | **Allele** | **Stage** | **Foci** |
| --- | --- | --- | --- | --- | --- | --- | --- | --- |
| WT | Zygotene | 189 | *atm-2* | Zygotene | 285 | *atm-5* | Zygotene | 264 |
| WT | Zygotene | 130 | *atm-2* | Zygotene | 209 | *atm-5* | Zygotene | 249 |
| WT | Zygotene | 200 | *atm-2* | Zygotene | 277 | *atm-5* | Zygotene | 212 |
| WT | Zygotene | 154 | *atm-2* | Zygotene | 275 | *atm-5* | Zygotene | 389 |
| WT | Zygotene | 168 | *atm-2* | Zygotene | 225 | *atm-5* | Zygotene | 237 |
| WT | Zygotene | 172 | *atm-2* | Zygotene | 252 | *atm-5* | Zygotene | 375 |
| WT | Zygotene | 144 | *atm-2* | Zygotene | 212 | *atm-5* | Zygotene | 210 |
| WT | Zygotene | 146 | *atm-2* | Zygotene | 225 | *atm-5* | Zygotene | 289 |
| WT | Zygotene | 147 | *atm-2* | Zygotene | 252 | *atm-5* | Zygotene | 302 |
| WT | Zygotene | 186 | *atm-2* | Zygotene | 262 | *atm-5* | Zygotene | 319 |
| WT | Zygotene | 174 | *atm-2* | Zygotene | 255 | *atm-5* | Zygotene | 251 |
| WT | Zygotene | 191 | *atm-2* | Zygotene | 213 | *atm-5* | Zygotene | 348 |
| WT | Zygotene | 184 | *atm-2* | Zygotene | 250 | *atm-5* | Zygotene | 289 |
| WT | Zygotene | 153 | *atm-2* | Zygotene | 269 | *atm-5* | Zygotene | 257 |
| WT | Zygotene | 177 | *atm-2* | Zygotene | 228 | *atm-5* | Zygotene | 305 |
| WT | Zygotene | 189 | *atm-2* | Zygotene | 353 | *atm-5* | Zygotene | 268 |
| WT | Zygotene | 188 | *atm-2* | Zygotene | 225 | *atm-5* | Zygotene | 237 |
| WT | Zygotene | 183 | *atm-2* | Zygotene | 254 | *atm-5* | Zygotene | 307 |
| WT | Zygotene | 162 | *atm-2* | Zygotene | 336 | *atm-5* | Zygotene | 265 |
| WT | Zygotene | 160 | *atm-2* | Zygotene | 345 | *atm-5* | Zygotene | 305 |
| WT | Zygotene | 158 | *atm-2* | Zygotene | 315 | *atm-5* | Zygotene | 313 |
| WT | Zygotene | 159 | *atm-2* | Zygotene | 205 | *atm-5* | Zygotene | 333 |
| WT | Zygotene | 142 | *atm-2* | Zygotene | 235 | *atm-5* | Zygotene | 225 |
| WT | Zygotene | 134 | *atm-2* | Zygotene | 380 | *atm-5* | Zygotene | 283 |
| WT | Zygotene | 149 | *atm-2* | Zygotene | 288 | *atm-5* | Zygotene | 281 |
| WT | Zygotene | 129 | *atm-2* | Zygotene | 358 | *atm-5* | Zygotene | 244 |
| WT | Zygotene | 135 | *atm-2* | Zygotene | 330 | *atm-5* | Zygotene | 277 |
|  |  |  | *atm-2* | Zygotene | 267 | *atm-5* | Zygotene | 310 |
|  |  |  | *atm-2* | Zygotene | 339 | *atm-5* | Zygotene | 244 |
|  |  |  | *atm-2* | Zygotene | 344 | *atm-5* | Zygotene | 259 |
|  |  |  | *atm-2* | Zygotene | 278 | *atm-5* | Zygotene | 269 |
|  |  |  | *atm-2* | Zygotene | 247 | *atm-5* | Zygotene | 246 |
|  |  |  | *atm-2* | Zygotene | 343 | *atm-5* | Zygotene | 318 |
|  |  |  | *atm-2* | Zygotene | 309 | *atm-5* | Zygotene | 382 |
|  |  |  | *atm-2* | Zygotene | 411 | *atm-5* | Zygotene | 279 |
|  |  |  | *atm-2* | Zygotene | 223 | *atm-5* | Zygotene | 343 |
|  |  |  | *atm-2* | Zygotene | 277 |  |  |  |
|  |  |  | *atm-2* | Zygotene | 289 |  |  |  |
|  |  |  | *atm-2* | Zygotene | 247 |  |  |  |
| **Allele** | **Stage** | **Foci** | **Allele** | **Stage** | **Foci** | **Allele** | **Stage** | **Foci** |
| WT | Pachytene | 31 | *atm-2* | Pachytene | 68 | *atm-5* | Pachytene | 49 |
| WT | Pachytene | 30 | *atm-2* | Pachytene | 76 | *atm-5* | Pachytene | 48 |
| WT | Pachytene | 29 | *atm-2* | Pachytene | 55 | *atm-5* | Pachytene | 49 |
| WT | Pachytene | 28 | *atm-2* | Pachytene | 65 | *atm-5* | Pachytene | 42 |
| WT | Pachytene | 25 | *atm-2* | Pachytene | 52 | *atm-5* | Pachytene | 57 |
| WT | Pachytene | 24 | *atm-2* | Pachytene | 68 | *atm-5* | Pachytene | 44 |
| WT | Pachytene | 24 | *atm-2* | Pachytene | 54 | *atm-5* | Pachytene | 46 |
| WT | Pachytene | 26 | *atm-2* | Pachytene | 37 | *atm-5* | Pachytene | 55 |
| WT | Pachytene | 34 | *atm-2* | Pachytene | 38 | *atm-5* | Pachytene | 66 |
| WT | Pachytene | 45 | *atm-2* | Pachytene | 53 | *atm-5* | Pachytene | 65 |
| WT | Pachytene | 30 | *atm-2* | Pachytene | 65 | *atm-5* | Pachytene | 54 |
| WT | Pachytene | 35 | *atm-2* | Pachytene | 43 | *atm-5* | Pachytene | 43 |
| WT | Pachytene | 30 | *atm-2* | Pachytene | 42 | *atm-5* | Pachytene | 50 |
| WT | Pachytene | 35 | *atm-2* | Pachytene | 62 | *atm-5* | Pachytene | 59 |
| WT | Pachytene | 27 | *atm-2* | Pachytene | 58 | *atm-5* | Pachytene | 49 |
| WT | Pachytene | 30 | *atm-2* | Pachytene | 71 | *atm-5* | Pachytene | 54 |
| WT | Pachytene | 40 | *atm-2* | Pachytene | 47 | *atm-5* | Pachytene | 60 |
| WT | Pachytene | 32 | *atm-2* | Pachytene | 53 | *atm-5* | Pachytene | 66 |
| WT | Pachytene | 34 | *atm-2* | Pachytene | 57 | *atm-5* | Pachytene | 48 |
| WT | Pachytene | 21 | *atm-2* | Pachytene | 61 | *atm-5* | Pachytene | 56 |
| WT | Pachytene | 32 | *atm-2* | Pachytene | 53 | *atm-5* | Pachytene | 56 |
| WT | Pachytene | 30 | *atm-2* | Pachytene | 76 | *atm-5* | Pachytene | 52 |
| WT | Pachytene | 28 | *atm-2* | Pachytene | 71 | *atm-5* | Pachytene | 36 |
| WT | Pachytene | 30 | *atm-2* | Pachytene | 73 | *atm-5* | Pachytene | 49 |
| WT | Pachytene | 28 | *atm-2* | Pachytene | 63 | *atm-5* | Pachytene | 56 |
| WT | Pachytene | 34 | *atm-2* | Pachytene | 75 | *atm-5* | Pachytene | 55 |
| WT | Pachytene | 28 | *atm-2* | Pachytene | 54 | *atm-5* | Pachytene | 50 |
| WT | Pachytene | 26 | *atm-2* | Pachytene | 67 | *atm-5* | Pachytene | 67 |
| WT | Pachytene | 39 | *atm-2* | Pachytene | 46 | *atm-5* | Pachytene | 69 |
| WT | Pachytene | 31 | *atm-2* | Pachytene | 63 | *atm-5* | Pachytene | 45 |
| WT | Pachytene | 41 | *atm-2* | Pachytene | 56 | *atm-5* | Pachytene | 63 |
| WT | Pachytene | 35 | *atm-2* | Pachytene | 60 | *atm-5* | Pachytene | 57 |
| WT | Pachytene | 44 | *atm-2* | Pachytene | 60 |  |  |  |
| WT | Pachytene | 32 | *atm-2* | Pachytene | 65 |  |  |  |
| WT | Pachytene | 44 | *atm-2* | Pachytene | 50 |  |  |  |
| WT | Pachytene | 51 | *atm-2* | Pachytene | 66 |  |  |  |
| WT | Pachytene | 34 | *atm-2* | Pachytene | 57 |  |  |  |
| WT | Pachytene | 29 | *atm-2* | Pachytene | 54 |  |  |  |
| WT | Pachytene | 31 | *atm-2* | Pachytene | 66 |  |  |  |
| WT | Pachytene | 34 | *atm-2* | Pachytene | 48 |  |  |  |
| WT | Pachytene | 26 | *atm-2* | Pachytene | 62 |  |  |  |
| WT | Pachytene | 36 | *atm-2* | Pachytene | 72 |  |  |  |
| WT | Pachytene | 35 | *atm-2* | Pachytene | 48 |  |  |  |
| WT | Pachytene | 26 |  |  |  |  |  |  |
| WT | Pachytene | 26 |  |  |  |  |  |  |
| WT | Pachytene | 38 |  |  |  |  |  |  |
| WT | Pachytene | 33 |  |  |  |  |  |  |
