# Supplementary material for: ATM Promotes RAD51-Mediated Meiotic DSB Repair by Inter-Sister-Chromatid Recombination in Arabidopsis
Source: Front Plant Sci. 2020 Jun 25;11:839. doi: 10.3389/fpls.2020.00839 (PMC7329986; doi:10.3389/fpls.2020.00839)
Supplement: TABLE S6 — Numbers of counted chiasmata in each image. [file Table_6.DOCX]

**Table S6. Numbers of counted chiasmata in each image.**

| **Allele** | **Total chiasmata** | **Each bivalent** | | | | | **Allele** | **Total chiasmata** | **Each bivalent** | | | | | **Allele** | **Total chiasmata** | **Each bivalent** | | | | |
| --- | --- | --- | --- | --- | --- | --- | --- | --- | --- | --- | --- | --- | --- | --- | --- | --- | --- | --- | --- | --- |
| WT | 9 | 3 | 1 | 2 | 1 | 2 | *atm-2* | 12 | 3 | 2 | 3 | 2 | 2 | *atm-5* | 11 | 2 | 2 | 3 | 2 | 2 |
| WT | 12 | 2 | 3 | 3 | 2 | 2 | *atm-2* | 11 | 3 | 2 | 1 | 3 | 2 | *atm-5* | 14 | 3 | 3 | 3 | 3 | 2 |
| WT | 10 | 2 | 3 | 1 | 2 | 2 | *atm-2* | 11 | 2 | 2 | 1 | 3 | 3 | *atm-5* | 12 | 3 | 3 | 2 | 3 | 1 |
| WT | 10 | 3 | 2 | 2 | 1 | 2 | *atm-2* | 10 | 2 | 3 | 2 | 2 | 1 | *atm-5* | 13 | 3 | 3 | 3 | 2 | 2 |
| WT | 11 | 3 | 2 | 2 | 2 | 2 | *atm-2* | 13 | 3 | 3 | 3 | 2 | 2 | *atm-5* | 10 | 3 | 2 | 1 | 2 | 2 |
| WT | 11 | 2 | 2 | 2 | 3 | 2 | *atm-2* | 11 | 1 | 2 | 3 | 3 | 2 | *atm-5* | 13 | 3 | 2 | 2 | 3 | 3 |
| WT | 13 | 3 | 2 | 3 | 2 | 3 | *atm-2* | 12 | 2 | 2 | 3 | 3 | 2 | *atm-5* | 11 | 2 | 3 | 1 | 3 | 2 |
| WT | 11 | 2 | 2 | 2 | 2 | 3 | *atm-2* | 10 | 2 | 2 | 2 | 2 | 2 | *atm-5* | 12 | 2 | 2 | 2 | 3 | 3 |
| WT | 11 | 2 | 2 | 3 | 3 | 1 | *atm-2* | 14 | 3 | 3 | 3 | 3 | 2 | *atm-5* | 14 | 3 | 3 | 3 | 3 | 2 |
| WT | 12 | 2 | 3 | 2 | 3 | 2 | *atm-2* | 13 | 2 | 3 | 3 | 3 | 2 | *atm-5* | 13 | 3 | 3 | 3 | 2 | 2 |
| WT | 10 | 2 | 1 | 3 | 2 | 2 | *atm-2* | 13 | 3 | 2 | 3 | 3 | 2 | *atm-5* | 10 | 2 | 2 | 2 | 2 | 2 |
| WT | 11 | 2 | 2 | 3 | 2 | 2 | *atm-2* | 12 | 2 | 3 | 3 | 2 | 2 | *atm-5* | 14 | 3 | 3 | 3 | 3 | 2 |
| WT | 9 | 2 | 1 | 1 | 3 | 2 | *atm-2* | 12 | 2 | 2 | 2 | 3 | 3 | *atm-5* | 12 | 3 | 2 | 3 | 2 | 2 |
| WT | 10 | 2 | 2 | 2 | 1 | 3 | *atm-2* | 11 | 3 | 2 | 2 | 3 | 1 | *atm-5* | 13 | 3 | 3 | 3 | 2 | 2 |
| WT | 12 | 3 | 2 | 2 | 2 | 3 | *atm-2* | 13 | 3 | 3 | 3 | 2 | 2 | *atm-5* | 12 | 3 | 2 | 3 | 2 | 2 |
| WT | 9 | 2 | 1 | 2 | 1 | 3 | *atm-2* | 13 | 3 | 2 | 2 | 3 | 3 | *atm-5* | 11 | 3 | 2 | 2 | 2 | 2 |
| WT | 12 | 3 | 2 | 2 | 2 | 3 | *atm-2* | 13 | 3 | 3 | 3 | 2 | 2 | *atm-5* | 11 | 1 | 2 | 2 | 3 | 3 |
| WT | 10 | 2 | 2 | 1 | 2 | 3 | *atm-2* | 12 | 2 | 3 | 2 | 3 | 2 | *atm-5* | 13 | 3 | 3 | 2 | 2 | 3 |
| WT | 12 | 2 | 3 | 2 | 2 | 3 | *atm-2* | 11 | 3 | 2 | 3 | 2 | 1 | *atm-5* | 11 | 2 | 2 | 2 | 2 | 3 |
| WT | 12 | 2 | 3 | 2 | 2 | 3 | *atm-2* | 13 | 3 | 2 | 2 | 3 | 3 | *atm-5* | 10 | 2 | 3 | 2 | 2 | 1 |
|  |  |  |  |  |  |  | *atm-2* | 13 | 3 | 2 | 3 | 2 | 3 | *atm-5* | 14 | 2 | 3 | 3 | 3 | 3 |
|  |  |  |  |  |  |  | *atm-2* | 12 | 3 | 2 | 3 | 2 | 2 |  |  |  |  |  |  |  |
|  |  |  |  |  |  |  | *atm-2* | 12 | 2 | 2 | 2 | 3 | 3 |  |  |  |  |  |  |  |
|  |  |  |  |  |  |  | *atm-2* | 11 | 2 | 2 | 2 | 2 | 3 |  |  |  |  |  |  |  |
|  |  |  |  |  |  |  | *atm-2* | 12 | 2 | 2 | 2 | 3 | 3 |  |  |  |  |  |  |  |
