# Supplementary material for: ATM Promotes RAD51-Mediated Meiotic DSB Repair by Inter-Sister-Chromatid Recombination in Arabidopsis
Source: Front Plant Sci. 2020 Jun 25;11:839. doi: 10.3389/fpls.2020.00839 (PMC7329986; doi:10.3389/fpls.2020.00839)
Supplement: TABLE S7 — Number of cells counted in each type of mutants. [file Table_7.DOCX]

**Table S7. Number of cells counted in each type of mutants**

The numbers of counted metaphase I cells containing different number of bivalents or univalent

|  | **5 bivalents** | **4 bivalents** | **3 bivalents** | **2 bivalents** | **1 bivalent** | **10 univalents** | **Entangled** | **Univalents and Entangled** |
| --- | --- | --- | --- | --- | --- | --- | --- | --- |
| Col | 33 | 0 | 0 | 0 | 0 | 0 | 0 | 0 |
| *atm-2* | 39 | 9 | 8 | 0 | 0 | 0 | 0 | 0 |
| *atm-5* | 27 | 11 | 0 | 0 | 0 | 0 | 0 | 0 |
| *dmc1* | 0 | 0 | 0 | 0 | 0 | 31 | 0 | 0 |
| *rad51-1* | 0 | 0 | 0 | 0 | 0 | 0 | 14 | 0 |
| *atm-5 dmc1* | 0 | 0 | 0 | 0 | 0 | 0 | 41 | 0 |
| *atm-5 rad51-1* | 0 | 0 | 0 | 0 | 0 | 0 | 36 | 0 |
| *asy1* | 0 | 1 | 1 | 6 | 10 | 7 | 0 | 0 |
| *atm-5 asy1* | 0 | 0 | 0 | 0 | 0 | 0 | 0 | 37 |

The numbers of counted metaphase II and telophase II cells containing different number of chromosome fragments

|  | **0** | **1-5** | **6-10** | **>10** |
| --- | --- | --- | --- | --- |
| Col | 42 | 0 | 0 | 0 |
| *atm-5* | 4 | 40 | 15 | 2 |
| *rad51-1* | 0 | 0 | 1 | 31 |
| *atm-5 rad51-1* | 0 | 0 | 0 | 31 |
| *dmc1* | 27 | 1 | 0 | 0 |
| *atm-5 dmc1* | 0 | 0 | 0 | 41 |
| *asy1* | 17 | 0 | 0 | 0 |
| *atm-5 asy1* | 0 | 0 | 3 | 26 |
