# Supplementary material for: ATM Promotes RAD51-Mediated Meiotic DSB Repair by Inter-Sister-Chromatid Recombination in Arabidopsis
Source: Front Plant Sci. 2020 Jun 25;11:839. doi: 10.3389/fpls.2020.00839 (PMC7329986; doi:10.3389/fpls.2020.00839)
Supplement: TABLE S8 — Number of counted HEI10 immunolocalization signal foci in each image. [file Table_8.DOCX]

**Table S8. Numbers of counted HEI10 immunolocalization signal foci in each image.**

| **Allele** | **Stage** | **Foci** | **Allele** | **Stage** | **Foci** | **Allele** | **Stage** | **Foci** |
| --- | --- | --- | --- | --- | --- | --- | --- | --- |
| WT | Pachytene | 13 | *atm-2* | Pachytene | 13 | *atm-5* | Pachytene | 13 |
| WT | Pachytene | 12 | *atm-2* | Pachytene | 9 | *atm-5* | Pachytene | 8 |
| WT | Pachytene | 12 | *atm-2* | Pachytene | 13 | *atm-5* | Pachytene | 9 |
| WT | Pachytene | 11 | *atm-2* | Pachytene | 14 | *atm-5* | Pachytene | 12 |
| WT | Pachytene | 11 | *atm-2* | Pachytene | 10 | *atm-5* | Pachytene | 10 |
| WT | Pachytene | 11 | *atm-2* | Pachytene | 9 | *atm-5* | Pachytene | 10 |
| WT | Pachytene | 11 | *atm-2* | Pachytene | 6 | *atm-5* | Pachytene | 11 |
| WT | Pachytene | 10 | *atm-2* | Pachytene | 14 | *atm-5* | Pachytene | 9 |
| WT | Pachytene | 10 | *atm-2* | Pachytene | 11 | *atm-5* | Pachytene | 9 |
| WT | Pachytene | 10 | *atm-2* | Pachytene | 7 | *atm-5* | Pachytene | 10 |
| WT | Pachytene | 10 | *atm-2* | Pachytene | 14 | *atm-5* | Pachytene | 10 |
| WT | Pachytene | 10 | *atm-2* | Pachytene | 12 | *atm-5* | Pachytene | 12 |
| WT | Pachytene | 10 | *atm-2* | Pachytene | 12 | *atm-5* | Pachytene | 10 |
| WT | Pachytene | 10 | *atm-2* | Pachytene | 12 | *atm-5* | Pachytene | 9 |
| WT | Pachytene | 10 | *atm-2* | Pachytene | 10 | *atm-5* | Pachytene | 11 |
| WT | Pachytene | 9 | *atm-2* | Pachytene | 13 | *atm-5* | Pachytene | 12 |
| WT | Pachytene | 9 | *atm-2* | Pachytene | 7 | *atm-5* | Pachytene | 11 |
| WT | Pachytene | 9 | *atm-2* | Pachytene | 15 | *atm-5* | Pachytene | 12 |
| WT | Pachytene | 9 | *atm-2* | Pachytene | 15 | *atm-5* | Pachytene | 10 |
| WT | Pachytene | 8 | *atm-2* | Pachytene | 14 | *atm-5* | Pachytene | 14 |
| WT | Pachytene | 8 | *atm-2* | Pachytene | 14 | *atm-5* | Pachytene | 10 |
| WT | Pachytene | 8 | *atm-2* | Pachytene | 9 | *atm-5* | Pachytene | 11 |
| WT | Pachytene | 7 | *atm-2* | Pachytene | 11 | *atm-5* | Pachytene | 12 |
| WT | Pachytene | 8 | *atm-2* | Pachytene | 12 | *atm-5* | Pachytene | 9 |
| WT | Pachytene | 10 | *atm-2* | Pachytene | 12 | *atm-5* | Pachytene | 13 |
| WT | Pachytene | 8 | *atm-2* | Pachytene | 13 | *atm-5* | Pachytene | 10 |
| WT | Pachytene | 11 | *atm-2* | Pachytene | 11 | *atm-5* | Pachytene | 12 |
| WT | Pachytene | 9 | *atm-2* | Pachytene | 11 | *atm-5* | Pachytene | 9 |
| WT | Pachytene | 10 | *atm-2* | Pachytene | 14 | *atm-5* | Pachytene | 11 |
| WT | Pachytene | 8 | *atm-2* | Pachytene | 11 | *atm-5* | Pachytene | 10 |
| WT | Pachytene | 9 | *atm-2* | Pachytene | 9 | *atm-5* | Pachytene | 9 |
| WT | Pachytene | 12 | *atm-2* | Pachytene | 12 | *atm-5* | Pachytene | 10 |
| WT | Pachytene | 8 | *atm-2* | Pachytene | 12 | *atm-5* | Pachytene | 13 |
| WT | Pachytene | 10 | *atm-2* | Pachytene | 8 | *atm-5* | Pachytene | 11 |
| WT | Pachytene | 10 | *atm-2* | Pachytene | 12 | *atm-5* | Pachytene | 12 |
| WT | Pachytene | 8 | *atm-2* | Pachytene | 9 | *atm-5* | Pachytene | 10 |
| WT | Pachytene | 9 | *atm-2* | Pachytene | 9 | *atm-5* | Pachytene | 10 |
| WT | Pachytene | 9 | *atm-2* | Pachytene | 12 | *atm-5* | Pachytene | 12 |
| WT | Pachytene | 9 | *atm-2* | Pachytene | 9 | *atm-5* | Pachytene | 12 |
| WT | Pachytene | 8 | *atm-2* | Pachytene | 12 | *atm-5* | Pachytene | 13 |
| WT | Pachytene | 8 | *atm-2* | Pachytene | 13 | *atm-5* | Pachytene | 10 |
| WT | Pachytene | 11 | *atm-2* | Pachytene | 11 | *atm-5* | Pachytene | 9 |
| WT | Pachytene | 10 | *atm-2* | Pachytene | 10 | *atm-5* | Pachytene | 11 |
| WT | Pachytene | 11 | *atm-2* | Pachytene | 10 | *atm-5* | Pachytene | 11 |
| WT | Pachytene | 12 | *atm-2* | Pachytene | 11 | *atm-5* | Pachytene | 12 |
| WT | Pachytene | 9 | *atm-2* | Pachytene | 9 | *atm-5* | Pachytene | 13 |
| WT | Pachytene | 9 | *atm-2* | Pachytene | 12 | *atm-5* | Pachytene | 11 |
| WT | Pachytene | 12 | *atm-2* | Pachytene | 11 | *atm-5* | Pachytene | 10 |
| WT | Pachytene | 10 | *atm-2* | Pachytene | 13 | *atm-5* | Pachytene | 14 |
| WT | Pachytene | 8 | *atm-2* | Pachytene | 14 | *atm-5* | Pachytene | 12 |
| WT | Pachytene | 10 | *atm-2* | Pachytene | 14 | *atm-5* | Pachytene | 12 |
| WT | Pachytene | 11 | *atm-2* | Pachytene | 14 | *atm-5* | Pachytene | 10 |
| WT | Pachytene | 11 | *atm-2* | Pachytene | 10 | *atm-5* | Pachytene | 12 |
| WT | Pachytene | 12 | *atm-2* | Pachytene | 10 | *atm-5* | Pachytene | 13 |
| WT | Pachytene | 9 | *atm-2* | Pachytene | 11 | *atm-5* | Pachytene | 12 |
| WT | Pachytene | 12 | *atm-2* | Pachytene | 9 | *atm-5* | Pachytene | 9 |
| WT | Pachytene | 8 | *atm-2* | Pachytene | 11 | *atm-5* | Pachytene | 10 |
| WT | Pachytene | 11 | *atm-2* | Pachytene | 11 | *atm-5* | Pachytene | 12 |
| WT | Pachytene | 9 | *atm-2* | Pachytene | 12 | *atm-5* | Pachytene | 14 |
| WT | Pachytene | 10 | *atm-2* | Pachytene | 12 | *atm-5* | Pachytene | 12 |
| WT | Pachytene | 8 | *atm-2* | Pachytene | 12 | *atm-5* | Pachytene | 13 |
| WT | Pachytene | 7 | *atm-2* | Pachytene | 9 | *atm-5* | Pachytene | 13 |
| WT | Pachytene | 9 | *atm-2* | Pachytene | 11 | *atm-5* | Pachytene | 10 |
| WT | Pachytene | 12 | *atm-2* | Pachytene | 10 | *atm-5* | Pachytene | 10 |
| WT | Pachytene | 10 | *atm-2* | Pachytene | 12 | *atm-5* | Pachytene | 10 |
| WT | Pachytene | 8 | *atm-2* | Pachytene | 11 |  |  |  |
| WT | Pachytene | 7 | *atm-2* | Pachytene | 14 |  |  |  |
| WT | Pachytene | 7 | *atm-2* | Pachytene | 13 |  |  |  |
| WT | Pachytene | 7 | *atm-2* | Pachytene | 11 |  |  |  |
| WT | Pachytene | 8 | *atm-2* | Pachytene | 11 |  |  |  |
| WT | Pachytene | 7 | *atm-2* | Pachytene | 11 |  |  |  |
| WT | Pachytene | 7 | *atm-2* | Pachytene | 12 |  |  |  |
| WT | Pachytene | 6 | *atm-2* | Pachytene | 12 |  |  |  |
| WT | Pachytene | 7 | *atm-2* | Pachytene | 11 |  |  |  |
| WT | Pachytene | 7 | *atm-2* | Pachytene | 10 |  |  |  |
| WT | Pachytene | 9 | *atm-2* | Pachytene | 10 |  |  |  |
| WT | Pachytene | 8 | *atm-2* | Pachytene | 9 |  |  |  |
| WT | Pachytene | 12 | *atm-2* | Pachytene | 11 |  |  |  |
| WT | Pachytene | 8 | *atm-2* | Pachytene | 11 |  |  |  |
| WT | Pachytene | 6 | *atm-2* | Pachytene | 11 |  |  |  |
| WT | Pachytene | 8 | *atm-2* | Pachytene | 13 |  |  |  |
| WT | Pachytene | 6 | *atm-2* | Pachytene | 9 |  |  |  |
| WT | Pachytene | 9 | *atm-2* | Pachytene | 14 |  |  |  |
| WT | Pachytene | 9 | *atm-2* | Pachytene | 11 |  |  |  |
| WT | Pachytene | 8 | *atm-2* | Pachytene | 15 |  |  |  |
| WT | Pachytene | 8 | *atm-2* | Pachytene | 10 |  |  |  |
|  |  |  | *atm-2* | Pachytene | 9 |  |  |  |
|  |  |  | *atm-2* | Pachytene | 11 |  |  |  |
|  |  |  | *atm-2* | Pachytene | 12 |  |  |  |
|  |  |  | *atm-2* | Pachytene | 9 |  |  |  |
|  |  |  | *atm-2* | Pachytene | 11 |  |  |  |
|  |  |  | *atm-2* | Pachytene | 12 |  |  |  |
|  |  |  | *atm-2* | Pachytene | 11 |  |  |  |
|  |  |  | *atm-2* | Pachytene | 10 |  |  |  |
|  |  |  | *atm-2* | Pachytene | 14 |  |  |  |
|  |  |  | *atm-2* | Pachytene | 10 |  |  |  |
|  |  |  | *atm-2* | Pachytene | 11 |  |  |  |
|  |  |  | *atm-2* | Pachytene | 12 |  |  |  |
|  |  |  | *atm-2* | Pachytene | 10 |  |  |  |
|  |  |  | *atm-2* | Pachytene | 14 |  |  |  |
|  |  |  | *atm-2* | Pachytene | 11 |  |  |  |
|  |  |  | *atm-2* | Pachytene | 13 |  |  |  |
|  |  |  | *atm-2* | Pachytene | 14 |  |  |  |
|  |  |  | *atm-2* | Pachytene | 10 |  |  |  |
|  |  |  | *atm-2* | Pachytene | 8 |  |  |  |
|  |  |  | *atm-2* | Pachytene | 12 |  |  |  |
|  |  |  | *atm-2* | Pachytene | 11 |  |  |  |
|  |  |  | *atm-2* | Pachytene | 12 |  |  |  |
|  |  |  | *atm-2* | Pachytene | 11 |  |  |  |
|  |  |  | *atm-2* | Pachytene | 12 |  |  |  |
|  |  |  | *atm-2* | Pachytene | 12 |  |  |  |
|  |  |  | *atm-2* | Pachytene | 11 |  |  |  |
|  |  |  | *atm-2* | Pachytene | 10 |  |  |  |
|  |  |  |  |  |  |  |  |  |
| Allele | Stage | Foci | Allele | Stage | Foci | Allele | Stage | Foci |
| WT | Diakinesis | 12 | *atm-2* | Diakinesis | 13 | *atm-5* | Diakinesis | 11 |
| WT | Diakinesis | 12 | *atm-2* | Diakinesis | 12 | *atm-5* | Diakinesis | 10 |
| WT | Diakinesis | 12 | *atm-2* | Diakinesis | 14 | *atm-5* | Diakinesis | 11 |
| WT | Diakinesis | 11 | *atm-2* | Diakinesis | 8 | *atm-5* | Diakinesis | 11 |
| WT | Diakinesis | 11 | *atm-2* | Diakinesis | 9 | *atm-5* | Diakinesis | 12 |
| WT | Diakinesis | 11 | *atm-2* | Diakinesis | 10 | *atm-5* | Diakinesis | 13 |
| WT | Diakinesis | 11 | *atm-2* | Diakinesis | 10 | *atm-5* | Diakinesis | 15 |
| WT | Diakinesis | 11 | *atm-2* | Diakinesis | 12 | *atm-5* | Diakinesis | 11 |
| WT | Diakinesis | 10 | *atm-2* | Diakinesis | 12 | *atm-5* | Diakinesis | 12 |
| WT | Diakinesis | 10 | *atm-2* | Diakinesis | 11 | *atm-5* | Diakinesis | 13 |
| WT | Diakinesis | 10 | *atm-2* | Diakinesis | 9 | *atm-5* | Diakinesis | 12 |
| WT | Diakinesis | 10 | *atm-2* | Diakinesis | 9 | *atm-5* | Diakinesis | 9 |
| WT | Diakinesis | 10 | *atm-2* | Diakinesis | 11 | *atm-5* | Diakinesis | 11 |
| WT | Diakinesis | 10 | *atm-2* | Diakinesis | 13 | *atm-5* | Diakinesis | 14 |
| WT | Diakinesis | 9 | *atm-2* | Diakinesis | 9 | *atm-5* | Diakinesis | 10 |
| WT | Diakinesis | 9 | *atm-2* | Diakinesis | 12 | *atm-5* | Diakinesis | 12 |
| WT | Diakinesis | 9 | *atm-2* | Diakinesis | 10 | *atm-5* | Diakinesis | 10 |
| WT | Diakinesis | 9 | *atm-2* | Diakinesis | 12 | *atm-5* | Diakinesis | 11 |
| WT | Diakinesis | 9 | *atm-2* | Diakinesis | 13 | *atm-5* | Diakinesis | 10 |
| WT | Diakinesis | 9 | *atm-2* | Diakinesis | 7 | *atm-5* | Diakinesis | 10 |
| WT | Diakinesis | 8 | *atm-2* | Diakinesis | 12 | *atm-5* | Diakinesis | 10 |
| WT | Diakinesis | 8 | *atm-2* | Diakinesis | 11 | *atm-5* | Diakinesis | 10 |
| WT | Diakinesis | 8 | *atm-2* | Diakinesis | 11 | *atm-5* | Diakinesis | 11 |
|  |  |  | *atm-2* | Diakinesis | 10 | *atm-5* | Diakinesis | 12 |
|  |  |  | *atm-2* | Diakinesis | 12 | *atm-5* | Diakinesis | 8 |
|  |  |  | *atm-2* | Diakinesis | 11 | *atm-5* | Diakinesis | 10 |
|  |  |  | *atm-2* | Diakinesis | 11 | *atm-5* | Diakinesis | 10 |
|  |  |  | *atm-2* | Diakinesis | 11 | *atm-5* | Diakinesis | 8 |
|  |  |  | *atm-2* | Diakinesis | 12 | *atm-5* | Diakinesis | 12 |
|  |  |  | *atm-2* | Diakinesis | 10 | *atm-5* | Diakinesis | 11 |
|  |  |  | *atm-2* | Diakinesis | 12 | *atm-5* | Diakinesis | 10 |
|  |  |  | *atm-2* | Diakinesis | 11 | *atm-5* | Diakinesis | 11 |
|  |  |  | *atm-2* | Diakinesis | 10 | *atm-5* | Diakinesis | 12 |
|  |  |  | *atm-2* | Diakinesis | 11 | *atm-5* | Diakinesis | 12 |
|  |  |  | *atm-2* | Diakinesis | 14 | *atm-5* | Diakinesis | 13 |
|  |  |  | *atm-2* | Diakinesis | 11 | *atm-5* | Diakinesis | 11 |
|  |  |  | *atm-2* | Diakinesis | 14 | *atm-5* | Diakinesis | 8 |
|  |  |  | *atm-2* | Diakinesis | 11 | *atm-5* | Diakinesis | 11 |
|  |  |  | *atm-2* | Diakinesis | 9 | *atm-5* | Diakinesis | 10 |
|  |  |  | *atm-2* | Diakinesis | 13 | *atm-5* | Diakinesis | 12 |
|  |  |  | *atm-2* | Diakinesis | 13 | *atm-5* | Diakinesis | 10 |
|  |  |  | *atm-2* | Diakinesis | 9 | *atm-5* | Diakinesis | 12 |
|  |  |  | *atm-2* | Diakinesis | 9 | *atm-5* | Diakinesis | 11 |
|  |  |  | *atm-2* | Diakinesis | 12 | *atm-5* | Diakinesis | 10 |
|  |  |  | *atm-2* | Diakinesis | 9 | *atm-5* | Diakinesis | 10 |
|  |  |  | *atm-2* | Diakinesis | 9 | *atm-5* | Diakinesis | 11 |
|  |  |  | *atm-2* | Diakinesis | 10 | *atm-5* | Diakinesis | 11 |
|  |  |  | *atm-2* | Diakinesis | 13 | *atm-5* | Diakinesis | 10 |
|  |  |  | *atm-2* | Diakinesis | 12 |  |  |  |
|  |  |  | *atm-2* | Diakinesis | 10 |  |  |  |
|  |  |  | *atm-2* | Diakinesis | 11 |  |  |  |
|  |  |  | *atm-2* | Diakinesis | 11 |  |  |  |
|  |  |  | *atm-2* | Diakinesis | 11 |  |  |  |
|  |  |  | *atm-2* | Diakinesis | 13 |  |  |  |
|  |  |  | *atm-2* | Diakinesis | 12 |  |  |  |
|  |  |  | *atm-2* | Diakinesis | 13 |  |  |  |
|  |  |  | *atm-2* | Diakinesis | 13 |  |  |  |
|  |  |  | *atm-2* | Diakinesis | 10 |  |  |  |
|  |  |  | *atm-2* | Diakinesis | 13 |  |  |  |
|  |  |  | *atm-2* | Diakinesis | 10 |  |  |  |
|  |  |  | *atm-2* | Diakinesis | 12 |  |  |  |
|  |  |  | *atm-2* | Diakinesis | 11 |  |  |  |
|  |  |  | *atm-2* | Diakinesis | 13 |  |  |  |
|  |  |  | *atm-2* | Diakinesis | 10 |  |  |  |
|  |  |  | *atm-2* | Diakinesis | 12 |  |  |  |
|  |  |  | *atm-2* | Diakinesis | 10 |  |  |  |
|  |  |  | *atm-2* | Diakinesis | 12 |  |  |  |
|  |  |  | *atm-2* | Diakinesis | 10 |  |  |  |
|  |  |  | *atm-2* | Diakinesis | 11 |  |  |  |
|  |  |  | *atm-2* | Diakinesis | 11 |  |  |  |
|  |  |  | *atm-2* | Diakinesis | 12 |  |  |  |
|  |  |  | *atm-2* | Diakinesis | 11 |  |  |  |
|  |  |  | *atm-2* | Diakinesis | 10 |  |  |  |
|  |  |  | *atm-2* | Diakinesis | 11 |  |  |  |
|  |  |  | *atm-2* | Diakinesis | 10 |  |  |  |
|  |  |  | *atm-2* | Diakinesis | 11 |  |  |  |
|  |  |  | *atm-2* | Diakinesis | 11 |  |  |  |
|  |  |  | *atm-2* | Diakinesis | 11 |  |  |  |
|  |  |  | *atm-2* | Diakinesis | 11 |  |  |  |
|  |  |  | *atm-2* | Diakinesis | 11 |  |  |  |
|  |  |  | *atm-2* | Diakinesis | 13 |  |  |  |
|  |  |  | *atm-2* | Diakinesis | 12 |  |  |  |
|  |  |  | *atm-2* | Diakinesis | 14 |  |  |  |
|  |  |  | *atm-2* | Diakinesis | 13 |  |  |  |
|  |  |  | *atm-2* | Diakinesis | 15 |  |  |  |
|  |  |  | *atm-2* | Diakinesis | 13 |  |  |  |
|  |  |  | *atm-2* | Diakinesis | 12 |  |  |  |
|  |  |  | *atm-2* | Diakinesis | 11 |  |  |  |
|  |  |  | *atm-2* | Diakinesis | 8 |  |  |  |
|  |  |  | *atm-2* | Diakinesis | 12 |  |  |  |
|  |  |  | *atm-2* | Diakinesis | 14 |  |  |  |
|  |  |  | *atm-2* | Diakinesis | 11 |  |  |  |
|  |  |  | *atm-2* | Diakinesis | 12 |  |  |  |
|  |  |  | *atm-2* | Diakinesis | 12 |  |  |  |
|  |  |  | *atm-2* | Diakinesis | 13 |  |  |  |
|  |  |  | *atm-2* | Diakinesis | 12 |  |  |  |
|  |  |  | *atm-2* | Diakinesis | 12 |  |  |  |
|  |  |  | *atm-2* | Diakinesis | 12 |  |  |  |
|  |  |  | *atm-2* | Diakinesis | 9 |  |  |  |
|  |  |  | *atm-2* | Diakinesis | 13 |  |  |  |
|  |  |  | *atm-2* | Diakinesis | 11 |  |  |  |
|  |  |  | *atm-2* | Diakinesis | 10 |  |  |  |
|  |  |  | *atm-2* | Diakinesis | 12 |  |  |  |
|  |  |  | *atm-2* | Diakinesis | 11 |  |  |  |
|  |  |  | *atm-2* | Diakinesis | 9 |  |  |  |
|  |  |  | *atm-2* | Diakinesis | 12 |  |  |  |
|  |  |  | *atm-2* | Diakinesis | 11 |  |  |  |
|  |  |  | *atm-2* | Diakinesis | 10 |  |  |  |
|  |  |  | *atm-2* | Diakinesis | 10 |  |  |  |
|  |  |  | *atm-2* | Diakinesis | 11 |  |  |  |
